# Coordinating a three-level contract farming supply chain with option contracts considering risk-averse farmer and retailer

**DOI:** 10.1371/journal.pone.0279115

**Published:** 2023-02-24

**Authors:** Changhua Liao, Qihui Lu, Li Lin

**Affiliations:** School of Business Administration, Zhejiang Gongshang University, Hangzhou, China; University of Belgrade Faculty of Organisational Sciences: Univerzitet u Beogradu Fakultet organizacionih nauka, SERBIA

## Abstract

We considered a three-level contract farming supply chain comprising a risk-averse farmer, a risk-neutral supplier, and a risk-averse retailer. The farmer plants and grows fresh agricultural products with yield uncertainty, the supplier is the leader of the supply chain and the designer of the contracts, and the retailer sells processed products with random demand. Under CVaR criterion, this paper discusses three option contracts between the supplier and the retailer, as well as wholesale price contracts or replenishment cost-sharing contracts between the supplier and the farmer. Results show that when the farmer is risk-neutral, option contracts with or without replenishment cost-sharing contracts can maximize the total profit and increase the profits of all members simultaneously. When the farmer and the retailer are risk-averse, only option contracts with replenishment cost-sharing contracts can ensure supply chain full coordination and Pareto improvement by adjusting the option parameters and making the farmer’s sharing ratio equal to his risk aversion coefficient. Moreover, through numerical analysis, we discovered that the interval of the Pareto improvement decreases with the retailer’s risk aversion coefficient and the quantity loss rate, and increases with the farmer’s risk aversion coefficient. The supplier will not be able to increase his own profits when the loss rate is excessively large. Therefore, the leader should consider the risk aversion degree of all parties and the quantity loss rate of fresh agricultural products before choosing contracts.

## 1 Introduction

Contract farming is a growing practice in both developed and developing countries. It generates necessary guarantees to sustain the continued operations of vulnerable farmers and the stable supply of agricultural products [1]. More than 60% of large farms in the United States have used contracts, covering roughly 40% of the annual value of agricultural products [2]. Moreover, in contrast to two main body transactions, three main body transactions more effectively deal with real world scenarios, such as breeding plants-slaughterhouses-meat markets and farmers-food processors-supermarkets. In this study, our research object is a farmer-supplier-retailer contract farming supply chain. The supplier signs a purchase contract with the farmer, with the supplier specifying the contract parameters. Then the farmer plants the initial fresh agricultural products (FAPs) according to the contract. During the planting process, the farmer usually faces yield uncertainty caused by uncontrollable factors such as extreme weather and natural disasters [3]. In addition to the challenges of yield uncertainty, the FAPs have a high loss rate in the distribution process [4]. This will exacerbate the uncertainty of the supply of FAPs in the entire channel circulation process. The supplier will also sign an order contract with the retailer who sells processed FAPs to customers. The supplier first processes the initial FAPs and sets the contract parameters. Then the retailer determines the order quantity according to the contract. The retailer cannot accurately quantify the actual market demand at this time, and thus faces demand uncertainty. Under the combined effect of the uncertain output of the farmer, the loss rate in the distribution process, and the uncertain demand of the retailer, a serious imbalance emerges between the supply and demand of FAPs in the entire supply chain, which will ultimately reduce the profits of all parties and supply chain.

Contract farming can solve the aforementioned problem and increase the supply chain’s profits. Ye et al. [5] indicated that the contract farming supply chain with stochastic yield and demand can be coordinated. The cooperation between supply chain members through various forms of contracts such as option contracts and cost-sharing contracts is the main reason for the success of contract farming. The option contract is an effective tool used to hedge supply chain risks and is widely used in the supply chain [6]. For example, China Telecom uses a call option contract in its purchases, which accounted for 100 billion RMB annually since 2009 [7]. It also represents 35% of HP’s procurement value in 2011 [6]. The use of such contracts in agriculture is still rare. Cost-sharing contract, which is a commonly used contract form, is used to share part of the other party’s uncertain risks by sharing costs [8]. It has many applications in agricultural supply chain [5, 9].

Numerous existing studies on contract farming assumed that the farmer is risk-neutral [1, 10]. However, to be more realistic, we considered that the farmer is risk-averse in the face of uncertainty in output. And the retailer with uncertain demand is also risk-averse. When facing the risk-averse farmer, the supplier, as the leader of the supply chain and contract designer, should provide the farmer with a contract that effectively shares his output risk and reduces his risk-averse factor. When facing the risk-averse retailer, the supplier should provide the retailer with what kind of contract. Ultimately, this research aims to promote the balance of supply and demand and optimize supply chain profits. Based on the background information described above, we aimed to study the following questions:

1. What type of contract would make the entire supply chain, including the risk-averse farmer, the leader supplier, and the risk-averse retailer, better off?
2. What are the effects of the loss rate, risk aversion coefficient, and option parameters on the decision of supply chain members?
3. How will supply chain members choose contracts?

To answer these questions, we considered a scenario where the supplier as the leader of the supply chain is the designer of the contract in the transaction between the supplier and the risk-averse retailer or the transaction between the risk-averse farmer and the supplier. Similar to Yang et al. [6], we used game theory method to analyze the coordination strategies with three options contracts, namely call options, put options and bidirectional options. The call option contract is the most commonly used tool to hedge the shortage risk, the put option contract is often used in the future market, and the bidirectional option contract can improve the flexibility of the supply chain. Each has its own advantages, so it is very necessary to make a comparative study of them. In addition, in order to coordinate the production behavior of the farmer, we also considered the cost-sharing contract.

To the best of our knowledge, this paper is the first study to incorporate simultaneously the yield and demand uncertain, the optimal decisions of farmer and retailer, three option contracts mechanisms, and the risk aversion of farmer and retailer into a three-level contract farming supply chain. Specifically, this study focuses on obtaining the conditions for full coordination and Pareto improvement of the supply chain in various scenarios and provide the selection preferences of supply chain members for contracts simultaneously. Our research uncovered the effects of the supply chain members’ cooperative mechanisms, the risk aversion coefficients, and the loss rates in distribution process of FAPs on their strategies, profits, and utilities under CVaR criterion. Moreover, we aim to promote the balance of supply and demand and optimize supply chain profits. These could provide managerial implications for the operational decisions of three-level contract farming supply chain and provide decision-making references for the leader supplier when facing risk-averse farmer and retailer.

The rest of the paper is organized as follows. We discussed the relevant literature and research gaps in Section 2. In Section 3, we defined our problem statements and formulated the models. In Section 4, we analyzed the optimal solutions in the cases of the centralized supply chain and the decentralized supply chain. In Section 5, we studied three option contracts between the supplier and the retailer, and considered the wholesale price contract or replenishment cost-sharing contract between the supplier and the farmer. In Section 6, we compared three option contracts and six scenarios to provide the selection preferences of the supply chain members for various contracts. Lastly, in Section 7, we discussed our contribution to the literature and drew managerial implications based on our results. All proofs are presented in Appendix B in S1 File.

## 2 Literature review

In this section, we reviewed the related literature, including FAP supply chain coordination, option contracts and cost-sharing contracts, and risk measure and analysis.

### 2.1 FAP supply chain coordination

In recent years, FAPs are receiving more attention, and numerous scholars have studied FAP issues in operations management [1, 10, 11]. Most of studies about coordination of FAP supply chain have focused only on two-level supply chain. For example, Moon et al. [12], Li et al. [13], Wan et al. [14], Zheng et al. [15], and Wang and Chen [16] explored the effectiveness of the revenue-sharing contract, profit-sharing contract, option contract, two-part tariff contract and combination contracts in the coordination process, respectively. Niu et al. [17] and Liu et al. [18] considered the effects of blockchain technology on the FAP two-level supply chain and realized supply chain coordination through coordination mechanism. There are fewer studies about the coordination of three-level FAP supply chain. Yan et al. [19] indicated that the improved revenue sharing contract can coordinate the three-level FAP supply chain that comprises a manufacturer, distributor, and retailer in the Internet of Things, which can benefit all enterprises in the supply chain. Song and He [9] considered a three-layer FAP supply chain that consists of a fresh produce e-commerce enterprise, third-party logistics service provider and community convenience store, and designed freshness-keeping cost-sharing and revenue-sharing contract. Similarly, Wu et al. [20] analyzed a two-part tariff contract. Ma et al. [21] designed a coordination contract based on cost and revenue sharing for the two transaction processes in the three-echelon FAP supply chain system consisting of one supplier, third-party logistics service providers, and one retailer. We also considered the coordination of the three-level FAP supply chain. Unlike them, we designed different option contracts and cost-sharing contracts in a risk-averse environment. Besides, we used the loss rate to reflect the characteristics of FAPs.

### 2.2 Option contracts and cost-sharing contracts

The supply chain contracts are used to induce one or more parties to make decisions that optimize the overall performance of the supply chain and improve the profit of each supply chain member [22]. Our paper discussed the option contracts and cost-sharing contracts. In terms of the option contracts, whether it is the common call option contract and put option contract, or the special bidirectional option contract, previous literature has clearly defined examples [6, 23–26]. Wan et al. [14] provided explicit option coordination conditions for a disrupted FAP supply chain under two supply chain structures. Wan and Chen [26] designed a feasible supply chain coordination strategy based on a combination of wholesale price and put option contracts to hedge against the risks of price and demand caused by inflation. Wang et al. [25] showed that a bidirectional option contract can reduce the negative impact of customer returns and enhance the firm’s profit, especially under high demand uncertainty. Yang et al. [6] introduced three coordinating option contracts led by the supplier to reduce the retailer’s risk (shortage, inventory, and bilateral risk) in a supplier-retailer agricultural supply chain. Similarly, we considered a combination of wholesale price contracts and three option contracts led by the supplier between the supplier and the retailer to reduce the retailer’s risk caused by uncertain demand. Simultaneously, we introduced a replenishment cost-sharing contract led by the supplier between the supplier and the farmer to reduce the farmer’s risk caused by uncertain yield. Some scholars considered the advertising cost, investment cost, and freshness-keeping cost sharing contract [9, 12, 27–29]. Arani et al. [30] introduced a novel mixed revenue-sharing option contract to coordinate a retailer manufacturer supply chain. In contrast to existing literature that applied these approaches to the two-level supply chain with risk neutral, we applied them to the three-level supply chain to explore whether the same coordination effect is achieved. Furthermore, we considered both the farmer and retailer to be risk averse.

### 2.3 Risk measure and analysis

Our work is also related to studies on risk measurement and analysis. Previous studies mainly used three criteria to quantify risk proneness and risk aversion in the supply chain: mean-variance (MV), value-at-risk (VaR), and conditional value-at-risk (CVaR). Zhuo et al. [31] studied the implications of risk considerations for option contracts in a two-echelon supply chain and investigated the conditions for coordinating the supply chain by using option contracts under the MV framework. Esmaeili-Najafabadi et al. [32] applied the VaR and the CVaR to analyze the risk-averse decision maker’s behavior, in which they indicated the impact of the decision maker’s attitude on the supplier selection and the order quantity. Our research uses the CVaR criterion to measure the farmer and the retailer’s risk aversion. The study stream of CVaR criterion started from Rockafellar and Uryasev [33, 34], in which CVaR and its minimization formula was first proposed. Following the fundamental properties of CVaR, the risk preferences of the decision maker expressed by CVaR have been examined by various scholars [3, 35, 36]. Specifically, Zhao et al. [36] investigated a supply chain consisting of a risk-neutral supplier and a risk-averse retailer, in which they found that with the call option contract, a distribution-free coordination condition was derived to achieve the Pareto improvement under CVaR criterion. We also analyzed the coordination effect of option contracts under CVaR criterion. The difference is that we considered a three-level FPA supply chain with a risk-averse farmer, a risk-neutral supplier, and a risk-averse retailer, in which the farmer faces a yield uncertainty and the retailer faces a demand uncertainty.

### 2.4 Research gap

In this subsection, we summarized the research gap between our study and the related literature and further highlighted our contributions. Our work is closely related to Yang et al. [6] and Ye et al. [5]. The paper and Yang et al. [6] all introduced three option contracts to reduce the retailer’s risk and considered the loss rate of FAPs in the distribution process. The difference is that we used the CVaR criterion to describe the risk-averse behavior of both the farmer and retailer in the three-level FAP supply chain. Ye et al. [5] also considered the coordination for contract farming supply chain with stochastic yield and demand under CVaR criterion. But, Ye et al. [5] mainly explored the coordination effect of cost-sharing and revenue-sharing contracts on the two-level supply chain, and they only considered that the farmer is risk-averse. In summary, this paper is the first study to incorporate simultaneously the yield and demand uncertain, three option contracts mechanisms, and the risk aversion of farmer and retailer into a three-level FAP supply chain. We intend to investigate how the supply chain members’ cooperative mechanisms, the risk aversion coefficients, and the loss rates in distribution process of FAPs affect their strategies, profits, and utilities under CVaR criterion. Furthermore, after introducing option contracts with and without replenishment cost sharing contracts, we obtained the conditions for full coordination and Pareto improvement of the supply chain in various scenarios and provide the selection preferences of supply chain members for contracts simultaneously. Realizing the risk aversion degree of all parties and the quantity loss rate of FAPs is beneficial to choosing and designing the appropriate contract.

## 3 Model description and assumptions

We considered a three-level contract-farming supply chain with a risk-averse farmer, a risk-averse retailer, and a supplier who orders the initial FAPs from the farmer and processes and distributes the products to the retailer. We assumed that a production yield uncertainty exists due to the weather and other unpredictable factors [37]. We supposed that the random output factor *y* is a nonnegative random variable characterized by the cumulative distribution function (CDF) *G*(*y*) and probability density function (PDF) *g*(*y*). In the selling season, the market demand *x* is uncertain, which is nonnegative and has a CDF *F*(*x*) and a PDF *f*(*x*). Due to the relatively stable environment of the production of finished products, we assumed that the supplier does not have production yield uncertainty in processing. We also assumed that each member of the supply chain is independent and symmetrical in information.

In the distribution periods, the FAPs may face losses in the distribution process, such as quantity and quality loss [4]. In this study, we considered the quantity loss. After the farmer’s output, in the process from the farmer’s loading to the arrival of the supplier, there is a loss caused by natural factors such as loading and unloading, packaging, squeezing, transportation, and so on. The loss rate is denoted as *β*_*s*_. After the supplier’s processing is completed, in the process after loading to when the product reaches the retailer, loss also exists, and *β*_*r*_ represents this loss rate. We supposed that the losses in distribution process are borne by the supplier, who is the leading player of the supply chain. In reality, the loss rate would most likely decrease along with the supply chain. We supposed that 0 < *β*_*r*_ < *β*_*s*_ < 1. To simplify the formula, we denoted $\Gamma \left(t\right)=\int_{0}^{(1-\beta_{r})t}xf(x)dx$.

Risk preference is the most important factor that influences the farmer’s and retailer’s decision-making. Thus, in this paper, we are focused on studying the behavior of the risk-averse farmer and retailer. A prevalent risk measurement in finance and operations management is the CVaR criterion [33, 34], which measures the average profit falling below the λ-quantile level and has good computational features. Denoting Π as the decision-maker’s random profit, we defined its λ-CVaR value as follows:

$$\begin{array}{c} \text{EU}=\max_{\alpha \in R}\{\alpha -\frac{1}{\lambda }E{\left[\alpha -\Pi \right]}^{+}\}, \end{array}$$

where [*x*]^+^ = max{*x*, 0}, *α* is a real number and represents the threshold of loss [35], λ represents risk aversion coefficient, λ ∈ (0, 1]. The smaller the value of λ, the more risk-averse the decision-maker would be. When λ = 1, the CVaR model degenerates into the risk-neutral model and *EU* = *E*(Π). Under the CVaR criterion, the decision maker’s goal is to minimize the downside risk of its random profit, that is, to maximize the value of CVaR.

Our coordination mechanism is related to option contracts. Normally, the option contract can be either the call option contract or the put option contract, which is characterized by two parameters, namely, the option price *o*_*i*_ and the exercise price *e*. For the call option contract, the option price *o*_1_ is an allowance paid by the retailer to the supplier for reserving one unit of the replenishment capacity and the exercise price *e* is the payment by the retailer to the supplier for exercising one unit of the call option. For the put option contract, the option price *o*_2_ is an allowance paid by the retailer to the supplier for canceling or returning one ordered unit of the product and the exercise price *e* is the refund from the supplier to the retailer for exercising one unit of the put option. When the retailer is unsure of the direction in which he will need to change his order quantity, the retailer can buy a bidirectional option at unit price *o*_3_, which provides him the right to adjust his initial order either upwards or downwards, depending on demand realization at the juncture when the option is exercised [6]. Moreover, the cost-sharing contracts are widely used by firms to mitigate or eliminate the double marginalization problem caused by asymmetric information of members in the supply chain [8]. We considered two scenarios with and without replenishment cost-sharing contracts between the supplier and the farmer. In a replenishment cost-sharing contract, the supplier assumes a percentage 1 − *φ* (0 ≤ *φ* < 1) of the farmer’s replenishment cost from the spot market to encourage the farmer to fulfill his own order requirements.

To ensure that the retailer, supplier, and farmer are willing to participate in the supply chain and the three types of option contracts are meaningful, we suppose *p*(1 − *β*_*r*_) > *e*(1 − *β*_*r*_) + *o*_3_ > *e*(1 − *β*_*r*_) + *o*_1_ > *w*_*r*_(1 − *β*_*r*_) > *e*(1 − *β*_*r*_) − *o*_2_ > *e*(1 − *β*_*r*_) − *o*_3_ and *w*_*f*_ > *c*_*f*_. To ensure that the retailer orders the initial quantity and option quantity, we suppose *e*(1 − *β*_*r*_) + *o*_1_ > *w*_*r*_(1 − *β*_*r*_) > *o*_1_ and *e*(1 − *β*_*r*_) + *o*_3_ > *w*_*r*_(1 − *β*_*r*_) > *o*_3_. To avoid the trivial case that the farmer never plants, we suppose *s*_*f*_ > *c*_*f*_. We summarized all notations of the paper Table 1 in Appendix A of S1 File.

## 4 Benchmark models

In the benchmark models, first the supplier provides wholesale price contracts to the farmer and retailer and sets the wholesale price (*w*_*f*_, *w*_*r*_). Then, the farmer decides a production input *R* on initial FAPs, the retailer determines an initial order quantity *Q* to the supplier, and the supplier provides the same order quantity *Q* of initial FAPs to the farmer. After that, the farmer invests in planting and delivers the initial FAPs to the supplier. The farmer will order the initial products from the spot market at a price *s*_*f*_ to fulfill the order when the farmer’s output cannot fulfill the supplier’s order quantity. Finally, the supplier delivers the products to the retailer after processing and the retailer sells them to customers at a sale price *p*.

### 4.1 Centralized supply chain (CD)

In a centralized setting, all supply chain agents (the supplier, the farmer, and the retailer) form a vertically integrated entity and a risk-neutral central planner decides the optimal production input (*R*_*T*_) and stocking level (*Q*_*T*_) for each channel. In such an integrated supply chain, the expected system-wide profit is given by,

$$\begin{array}{c} E\Pi_{T}^{CD}=E[p\;\text{min}\left[\left(1-\beta_{r}\right)Q_{T},x\right]-c_{s}Q_{T}-c_{f}R_{T}-s_{f}{\left(\frac{Q_{T}}{1-\beta_{s}}-yR_{T}\right)}^{+}]\\ =p\left(1-\beta_{r}\right)Q_{T}-p\int_{0}^{\left(1-\beta_{r}\right)Q_{T}}F(x)dx-c_{s}Q_{T}-c_{f}R_{T}-s_{f}R_{T}\int_{0}^{\frac{Q_{T}}{R_{T}\left(1-\beta_{s}\right)}}G(y)dy. \end{array}$$

The first term is the total revenue from sales. The second and third terms are costs for processing and planting the initial FAPs, respectively. The fourth term is the cost of buying initial FAPs from the spot market in the case of a low yield. The optimal decision for a centralized supply chain is described below in Proposition 1.

**Proposition 1** $E\Pi_{T}^{CD}$ *is jointly concave with* *Q*_*T*_ *and* *R*_*T*_, *and optimal* $\left(Q_{T}^{*},R_{T}^{*}\right)$ *are uniquely solved by*

$$\begin{array}{c} Q_{T}^{*}=\frac{1}{1-\beta_{r}}F^{-1}(1-\frac{c_{s}+c_{\eta }}{p(1-\beta_{r})}), \end{array}$$

$$\begin{array}{c} \int_{0}^{\eta }yg(y)dy=\frac{c_{f}}{s_{f}}, \end{array}$$ (1)

*where* $\eta =\frac{Q_{T}^{*}}{R_{T}^{*}\left(1-\beta_{s}\right)}$, $c_{\eta }=\frac{s_{f}}{1-\beta_{s}}G\left(\eta \right)$.

To ensure that $Q_{T}^{*}$ is meaningful, we supposed *p*(1 − *β*_*r*_) > *c*_*s*_ + *c*_*η*_.

### 4.2 Decentralized supply chain (DD)

First, in a decentralized setup, we considered the retailer’s ordering problem and the random profit function of the retailer is expressed as follows: $\Pi_{r}^{DD}=p\;\text{min}\left[\left(1-\beta_{r}\right)Q_{0},x\right]-w_{r}\left(1-\beta_{r}\right)Q_{0}$. The utility function of the risk aversion retailer under the CVaR criterion is $EU_{r}^{DD}=\alpha_{r}^{DD}-\frac{1}{\lambda_{r}}\int_{0}^{\left(1-\beta_{r}\right)Q_{0}}{\left[\alpha_{r}^{DD}+w_{r}\left(1-\beta_{r}\right)Q_{0}-px\right]}^{+}f\left(x\right)dx-\frac{1}{\lambda_{r}}\int_{\left(1-\beta_{r}\right)Q_{0}}^{+\infty }{\left[\alpha_{r}^{DD}-\left(p-w_{r}\right)\left(1-\beta_{r}\right)Q_{0}\right]}^{+}f\left(x\right)dx$.

Then, we considered the farmer’s planting planning problem. The farmer’s profit function is $\Pi_{f}^{DD}=\frac{Q_{0}w_{f}}{1-\beta_{s}}-c_{f}R_{0}-s_{f}{\left(\frac{Q_{0}}{1-\beta_{s}}-yR_{0}\right)}^{+}$. The first term is the revenue that the farmer receives from selling initial FAPs to the supplier, the second term is the farmer’s planting cost, and the third term is the farmer’s cost of buying initial FAPs from the spot market in the case of a low yield. And his utility function is $EU_{f}^{DD}=\alpha_{f}^{DD}-\frac{1}{\lambda_{f}}\int_{0}^{\frac{Q_{0}}{R_{0}\left(1-\beta_{s}\right)}}{\left(\alpha_{f}^{DD}-\frac{Q_{0}w_{f}}{1-\beta_{s}}+c_{f}R_{0}+\frac{s_{f}Q_{0}}{1-\beta_{s}}-s_{f}R_{0}y\right)}^{+}g\left(y\right)dy-\frac{1}{\lambda_{f}}\int_{\frac{Q_{0}}{R_{0}\left(1-\beta_{s}\right)}}^{+\infty }{\left(\alpha_{f}^{DD}-\frac{Q_{0}w_{f}}{1-\beta_{s}}+c_{f}R_{0}\right)}^{+}g\left(y\right)dy$. The equilibrium for a decentralized supply chain is described below in Proposition 2.

**Proposition 2** *In the decentralized supply chain, there are*

1. *For the retailer, the optimal threshold of loss is* $\alpha_{r}^{DD}=\left(p-w_{r}\right)\left(1-\beta_{r}\right)Q_{0}$, *and the optimal initial order quantity is* $Q_{0}^{*}=\frac{1}{1-\beta_{r}}F^{-1}(\frac{\lambda_{r}\left(p-w_{r}\right)}{p})$.
2. *For the farmer, the optimal threshold of loss is* $\alpha_{f}^{DD}=\frac{Q_{0}w_{f}}{1-\beta_{s}}-c_{f}R_{0}$, *and the unique optimal* $R_{0}^{*}$ *satisfies the following equation, where* $\eta_{1}=\frac{Q_{0}^{*}}{R_{0}^{*}\left(1-\beta_{s}\right)}$,
  $$\begin{array}{c} \int_{0}^{\eta_{1}}yg(y)dy=\frac{\lambda_{f}c_{f}}{s_{f}}. \end{array}$$ (2)

Thus, the retailer’s maximum expected profit and utility are $E\Pi_{r}^{DD*}=p\Gamma \left(Q_{0}^{*}\right)+\left(1-\lambda_{r}\right)\left(p-w_{r}\right)\left(1-\beta_{r}\right)Q_{0}^{*}$ and $EU_{r}^{DD*}=\frac{p}{\lambda_{r}}\Gamma \left(Q_{0}^{*}\right)$, respectively. The farmer’s optimal expected profit and utility are as follows:

$$\begin{array}{c} E\Pi_{f}^{DD*}=[w_{f}-s_{f}G\left(\eta_{1}\right)-\frac{\left(1-\lambda_{f}\right)c_{f}}{\eta_{1}}]\frac{Q_{0}^{*}}{1-\beta_{s}},\\ EU_{f}^{DD*}=[w_{f}-\frac{s_{f}G\left(\eta_{1}\right)}{\lambda_{f}}]\frac{Q_{0}^{*}}{1-\beta_{s}}. \end{array}$$ (3)

The supplier’s optimal expected profit is $E\Pi_{s}^{DD*}=[w_{r}\left(1-\beta_{r}\right)-\frac{w_{f}}{1-\beta_{s}}-c_{s}]Q_{0}^{*}$.

**Proposition 3** *In the decentralized supply chain, there are*

1. *If* λ_*f*_ = 1, *then* *η*_1_ = *η*, $Q_{0}^{*}<Q_{T}^{*}$ *and* $R_{0}^{*}<R_{T}^{*}$.
2. *If* 0 < λ_*f*_ < 1, *then* $\frac{Q_{0}^{*}}{R_{0}^{*}}<\frac{Q_{T}^{*}}{R_{T}^{*}}$.

Proposition 3(1) shows that the order quantity and production input under the decentralized decision are always smaller than those under the centralized decision when the farmer is risk-neutral. Proposition 3(2) shows that the order quantity and production input under the decentralized decision can never be simultaneously equal to those under the centralized decision when the farmer is risk-averse, that is, the conditions for full coordination cannot be reached. Therefore, we determined that the wholesale-price-only contract cannot fully coordinate the three-level FAP supply chain with uncertain output and random demand when the retailer is risk-averse and the farmer is risk-averse or risk-neutral. This supply chain coordinating options contracts are represented by the following section.

## 5 Three option contracts

In the option contracts, first the supplier provides the wholesale price contract with option contracts to the retailer, provides the wholesale price contract or with the replenishment cost-sharing contract to the farmer, and sets the wholesale price (*w*_*f*_, *w*_*r*_), option price *o*_*i*_(*i* = 1, 2, 3), exercise price *e*, and the farmer’s replenishment cost sharing ratio *φ*. Then the farmer decides a production input *R* on initial FAPs, the retailer determines the initial order quantity *Q* and option order quantity *q*, and the supplier provides the same total order quantity *Q* + *q* of initial FAPs to the farmer. The farmer then invests in planting and delivers the initial FAPs to the supplier. The farmer will order initial products from the spot market at a price of *s*_*f*_ to fulfill the order or will execute replenishment cost-sharing contract when the farmer’s output cannot fulfill the supplier’s total order quantity. Lastly, the supplier supplies the products to the retailer after processing, the retailer sells them to customers at a sale price *p* and will decide whether to execute the option contract.

### 5.1 Call option contracts

#### 5.1.1 Call option contract with wholesale price contract (CO)

In this scenario, the retailer can execute call options to replenish at a price of *e* from the supplier when the actual market demand exceeds initial order quantity *Q*_1_. Thus, the retailer’s profit is represented by $\Pi_{r}^{CO}=p\;\text{min}\left[\left(1-\beta_{r}\right)\left(Q_{1}+q_{1}\right),x\right]-o_{1}q_{1}-w_{r}\left(1-\beta_{r}\right)Q_{1}-e\;\text{min}\left[{\left[x-\left(1-\beta_{r}\right)Q_{1}\right]}^{+},\left(1-\beta_{r}\right)q_{1}\right]$. The first term is the retailer’s sales revenue, the second term is the retailer’s option ordering cost, the third term is the retailer’s initial ordering cost, and the fourth term is the cost of exercising call options. The utility function of the risk aversion retailer under the CVaR criterion is

$$\begin{array}{c} EU_{r}^{CO}=\alpha_{r}^{CO}-\frac{1}{\lambda_{r}}\int_{0}^{\left(1-\beta_{r}\right)Q_{1}}{\left[\alpha_{r}^{CO}+w_{r}\left(1-\beta_{r}\right)Q_{1}+o_{1}q_{1}-px\right]}^{+}f\left(x\right)dx\\ -\frac{1}{\lambda_{r}}\int_{\left(1-\beta_{r}\right)Q_{1}}^{\left(1-\beta_{r}\right)\left(Q_{1}+q_{1}\right)}{\left[\alpha_{r}^{CO}-\left(e-w_{r}\right)\left(1-\beta_{r}\right)Q_{1}+o_{1}q_{1}-\left(p-e\right)x\right]}^{+}f\left(x\right)dx\\ -\frac{1}{\lambda_{r}}\int_{\left(1-\beta_{r}\right)\left(Q_{1}+q_{1}\right)}^{+\infty }{\left[\alpha_{r}^{CO}-\left(p-w_{r}\right)\left(1-\beta_{r}\right)Q_{1}-\left(p-e\right)\left(1-\beta_{r}\right)q_{1}+o_{1}q_{1}\right]}^{+}f\left(x\right)dx. \end{array}$$ (4)

Then, the farmer’s profit is shown by $\Pi_{f}^{CO}=\frac{\left(Q_{1}+q_{1}\right)w_{f}}{1-\beta_{s}}-c_{f}R_{1}-s_{f}{\left(\frac{Q_{1}+q_{1}}{1-\beta_{s}}-yR_{1}\right)}^{+}$ and his utility function is $EU_{f}^{CO}=\alpha_{f}^{CO}-\frac{1}{\lambda_{f}}\int_{0}^{\frac{Q_{1}+q_{1}}{R_{1}\left(1-\beta_{s}\right)}}{\left[\alpha_{f}^{CO}-\frac{\left(Q_{1}+q_{1}\right)w_{f}}{1-\beta_{s}}+c_{f}R_{1}+\frac{s_{f}\left(Q_{1}+q_{1}\right)}{1-\beta_{s}}-s_{f}R_{1}y\right]}^{+}g\left(y\right)dy-\frac{1}{\lambda_{f}}\int_{\frac{Q_{1}+q_{1}}{R_{1}\left(1-\beta_{s}\right)}}^{+\infty }{\left[\alpha_{f}^{CO}-\frac{\left(Q_{1}+q_{1}\right)w_{f}}{1-\beta_{s}}+c_{f}R_{1}\right]}^{+}g\left(y\right)dy$. The equilibrium for a decentralized supply chain with the call option contract is described below in Proposition 4.

**Proposition 4** *In the call option contract, suppose* (*p* − *e*)(1 − *β*_*r*_)*w*_*r*_ > *po*_1_, *there are*

1. *For the retailer, the optimal threshold of loss is* $\alpha_{r}^{CO}=\left(p-w_{r}\right)\left(1-\beta_{r}\right)Q_{1}+\left(p-e\right)\left(1-\beta_{r}\right)q_{1}-o_{1}q_{1}$. $EU_{r}^{CO}$ *is jointly concave with* *Q*_1_ *and* *q*_1_, *and optimal* $\left(Q_{1}^{*},q_{1}^{*}\right)$ *are uniquely solved by*
  $$\begin{array}{c} Q_{1}^{*}=\frac{1}{1-\beta_{r}}F^{-1}(\frac{\lambda_{r}\left(\left(e-w_{r}\right)\left(1-\beta_{r}\right)+o_{1}\right)}{e(1-\beta_{r})})\\ q_{1}^{*}=\frac{1}{1-\beta_{r}}F^{-1}(\frac{\lambda_{r}\left(\left(p-e\right)\left(1-\beta_{r}\right)-o_{1}\right)}{\left(p-e\right)\left(1-\beta_{r}\right)})-\frac{1}{1-\beta_{r}}F^{-1}(\frac{\lambda_{r}\left(\left(e-w_{r}\right)\left(1-\beta_{r}\right)+o_{1}\right)}{e(1-\beta_{r})}). \end{array}$$
2. *For the farmer, the optimal threshold of loss is* $\alpha_{f}^{CO}=\frac{\left(Q_{1}+q_{1}\right)w_{f}}{1-\beta_{s}}-c_{f}R_{1}$, *and the unique optimal* $R_{1}^{*}$ *satisfies*
  $$\begin{array}{c} \int_{0}^{\frac{Q_{1}^{*}+q_{1}^{*}}{R_{1}^{*}\left(1-\beta_{s}\right)}}yg(y)dy=\frac{\lambda_{f}c_{f}}{s_{f}}. \end{array}$$ (5)

From Proposition 4(1), $Q_{1}^{*}<Q_{0}^{*}<Q_{1}^{*}+q_{1}^{*}$, and the retailer’s maximum expected profit and utility are $E\Pi_{r}^{CO*}=\left(p-e\right)\Gamma \left(Q_{1}^{*}+q_{1}^{*}\right)+e\Gamma \left(Q_{1}^{*}\right)+\left(1-\lambda_{r}\right)\left[\left(1-\beta_{r}\right)\left[\left(p-w_{r}\right)Q_{1}^{*}+\left(p-e\right)q_{1}^{*}\right]-o_{1}q_{1}^{*}\right]$ and $EU_{r}^{CO*}=\frac{p-e}{\lambda_{r}}\Gamma \left(Q_{1}^{*}+q_{1}^{*}\right)+\frac{e}{\lambda_{r}}\Gamma \left(Q_{1}^{*}\right)+\lambda_{r}\left[\left(1-\beta_{r}\right)\left[\left(p-w_{r}\right)Q_{1}^{*}+\left(p-e\right)q_{1}^{*}\right]-o_{1}q_{1}^{*}\right]$, respectively. From Eqs (2) and (5), $\eta_{1}=\frac{Q_{0}^{*}}{R_{0}^{*}\left(1-\beta_{s}\right)}=\frac{Q_{1}^{*}+q_{1}^{*}}{R_{1}^{*}\left(1-\beta_{s}\right)}$. Thus, the farmer’s optimal expected profit and utility are as follows:

$$\begin{array}{c} E\Pi_{f}^{CO*}=[w_{f}-s_{f}G\left(\eta_{1}\right)-\frac{\left(1-\lambda_{f}\right)c_{f}}{\eta_{1}}]\frac{Q_{1}^{*}+q_{1}^{*}}{1-\beta_{s}},\\ EU_{f}^{CO*}=[w_{f}-\frac{s_{f}G\left(\eta_{1}\right)}{\lambda_{f}}]\frac{Q_{1}^{*}+q_{1}^{*}}{1-\beta_{s}}. \end{array}$$ (6)

From Eqs (3) and (6), $E\Pi_{f}^{CO*}>E\Pi_{f}^{DD*}$ and $EU_{f}^{CO*}>EU_{f}^{DD*}$. These results show that after adding the call option contract, the farmer’s optimal expected profit and utility will be improved. The supplier’s profit function is $\Pi_{s}^{CO}=o_{1}q_{1}+w_{r}\left(1-\beta_{r}\right)Q_{1}+e\;\text{min}\left[\left(1-\beta_{r}\right)q_{1},{\left[x-\left(1-\beta_{r}\right)Q_{1}\right]}^{+}\right]-\frac{\left(Q_{1}+q_{1}\right)w_{f}}{1-\beta_{s}}-c_{s}\left(Q_{1}+q_{1}\right)$. Thus, in the call option contract with the wholesale price contract, the supplier’s optimal expected profit is $E\Pi_{s}^{CO*}=e\left[\Gamma \left(Q_{1}^{*}+q_{1}^{*}\right)-\Gamma \left(Q_{1}^{*}\right)\right]+(\frac{p\lambda_{r}o_{1}}{p-e}-\frac{w_{f}}{1-\beta_{s}}-c_{s})\left(Q_{1}^{*}+q_{1}^{*}\right)+\left(1-\lambda_{r}\right)\left[\left(w_{r}Q_{1}^{*}+eq_{1}^{*}\right)\left(1-\beta_{r}\right)+o_{1}q_{1}^{*}\right]$.

**Proposition 5** *In the call option contract, there are*

1. *If* λ_*f*_ = 1, $o_{1}=\frac{\left(p-e\right)[c_{s}+c_{\eta }-p\left(1-\beta_{r}\right)\left(1-\lambda_{r}\right)]}{\lambda_{r}p}$, *and* $\frac{w_{r}\left(1-\beta_{r}\right)-o_{1}}{1-\beta_{r}}<e<\frac{p[w_{r}\left(1-\beta_{r}\right)-o_{1}]}{w_{r}\left(1-\beta_{r}\right)}$, *then* $Q_{1}^{*}+q_{1}^{*}=Q_{T}^{*}$ *and* $R_{1}^{*}=R_{T}^{*}$.
2. *If* 0 < λ_*f*_ < 1, *then for any* *o*_1_ and *e*, *that is* $\frac{Q_{1}^{*}+q_{1}^{*}}{R_{1}^{*}}<\frac{Q_{T}^{*}}{R_{T}^{*}}$.

**Fig 1:**
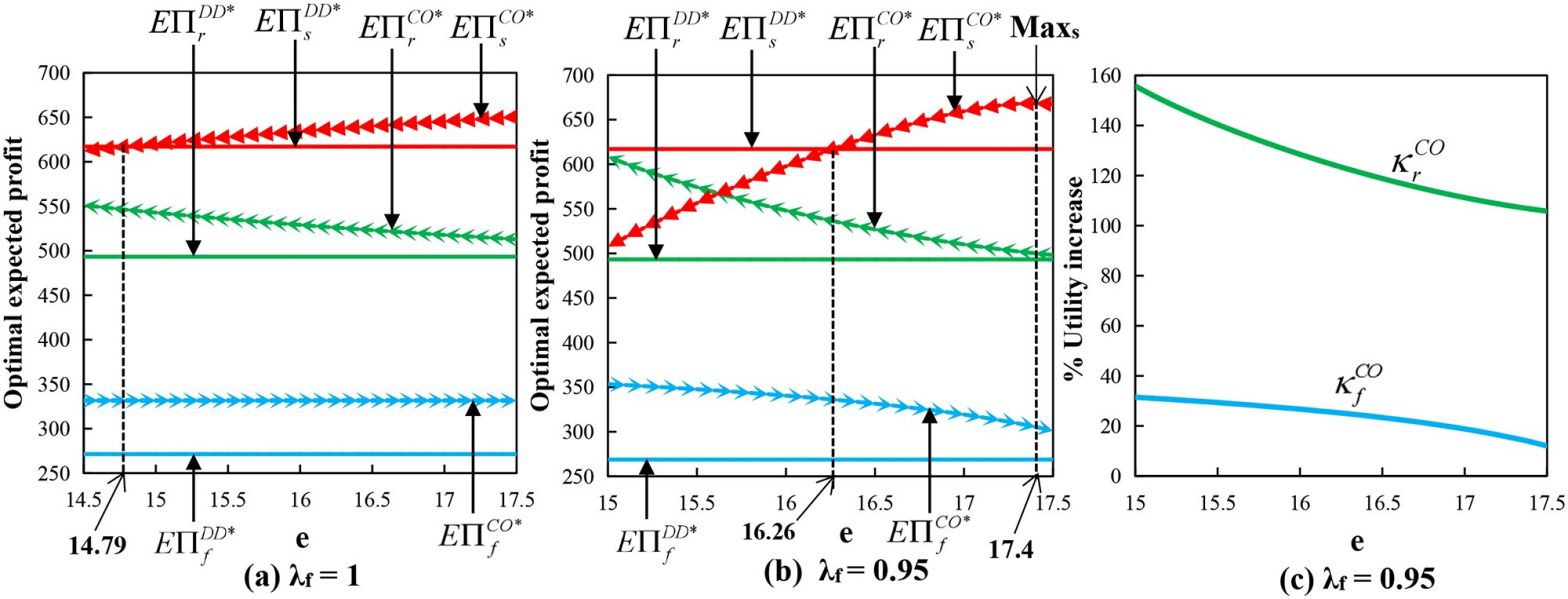
Impact of option exercise price under call option contract with wholesale price contract.

Proposition 5(1) represents that when the farmer is risk-neutral and (*o*_1_, *e*) meets a certain relationship condition, the supply chain can be fully coordinated. Moreover, Fig 1 provides the numerical results on the effect of the option exercise price on the optimal expected profits and utilities of each member, and we set the following parameter values: *x* and *y* all follow a Normal Distribution, *μ* = 200, *σ* = 40, *μ*_1_ = 1, *σ*_1_ = 0.2, *p* = 20, λ_*r*_ = 0.9, *β*_*r*_ = 0.1, *β*_*s*_ = 0.2, *c*_*s*_ = 2, *w*_*r*_ = 16.5, *c*_*f*_ = 6, *s*_*f*_ = 7.1, *w*_*f*_ = 7.5, and *o*_1_ = 1.5. Fig 1(a) shows that the supply chain can also achieve Pareto improvement in the interval of (14.79, 17.5). Proposition 5(2) represents that the order quantity and production input under the call option contract can never be equal to those under the centralized decision at the same time when the farmer is risk-averse with any *o*_1_ and *e*, in other words, the conditions for full coordination cannot be reached. However, Fig 1(b) shows that the supply chain can achieve the Pareto improvement in the interval of (16.26, 17.5).

From Fig 1(a) and 1(b), we determined whether the supply chain can be fully coordinated, the retailer’s optimal expected profit decreases with *e*, the supplier’s optimal expected profit increases first and then decreases with *e*, the supplier’s profit is maximized when *e* = 17.4, the farmer’s optimal expected profit decreases with *e* when the farmer has risk aversion and is independent on *e* when the farmer is risk-neutral. When the farmer has risk aversion, he will increase the production input to deal with the risks of spot price fluctuations and output uncertainty. At the same time, when *o* is given and *e* increases, the retailer will reduce the total order quantity and the supplier will also reduce the order quantity accordingly, which may lead to increasing the farmer’s production costs and overproduction and ultimately lower his profits. Furthermore, the retailer and the farmer’s expected profit are always greater than that under the decentralized decision, and whether Pareto improvement can be achieved depends on the change in the profit of the supplier.

In Fig 1(c), $\kappa_{r}^{CO}=\frac{EU_{r}^{CO*}-EU_{r}^{DD*}}{EU_{r}^{DD*}}*100\%$ represents the retailer’s optimal utility increase ratio and $\kappa_{f}^{CO}=\frac{EU_{f}^{CO*}-EU_{f}^{DD*}}{EU_{f}^{DD*}}*100\%$ represents the farmer’s optimal utility increase ratio in the call option contract. We determined that the optimal utility of the retailer and the farmer in the call option contract is increased compared to a decentralized decision. However, the increase rate decreases with the increase of call option exercise price. Therefore, whether it is profit or utility, the smaller exercise price is better for the retailer and farmer when given the option price.

#### 5.1.2 Call option contract with replenishment cost-sharing contract (CC)

When the retailer and farmer are risk-averse, to achieve the supply chain’s full coordination, we considered a replenishment cost-sharing contract in the transaction between the supplier and farmer. They share the cost of buying initial FAPs from the farmer’s spot market in the case of a low yield to reduce the effect of the spot price and hedge the degree of the farmer’s risk aversion. The farmer shares the replenishment cost ratio *φ*, and the supplier shares the replenishment cost ratio 1 − *φ*. The transaction between the supplier and farmer with or without the replenishment cost-sharing contract has no effect on the retailer’s equilibrium outcome, including the optimal initial order quantity and call option order quantity. Thus, $E\Pi_{r}^{CC*}=E\Pi_{r}^{CO*}$, and we can obtain the farmer’s profit function $\Pi_{f}^{CC}=\frac{\left(Q_{1}+q_{1}\right)w_{f}}{1-\beta_{s}}-c_{f}R_{1}^{C}-\phi s_{f}{\left(\frac{Q_{1}+q_{1}}{1-\beta_{s}}-yR_{1}^{C}\right)}^{+}$. Then, the utility function of the risk-averse farmer is $EU_{f}^{CC}=\alpha_{f}^{CC}-\frac{1}{\lambda_{f}}\int_{0}^{\frac{Q_{1}+q_{1}}{R_{1}^{C}\left(1-\beta_{s}\right)}}{\left[\alpha_{f}^{CC}-\frac{\left(Q_{1}+q_{1}\right)w_{f}}{1-\beta_{s}}+c_{f}R_{1}^{C}+\frac{\phi s_{f}\left(Q_{1}+q_{1}\right)}{1-\beta_{s}}-\phi s_{f}R_{1}^{C}y\right]}^{+}g\left(y\right)dy-\frac{1}{\lambda_{f}}\int_{\frac{Q_{1}+q_{1}}{R_{1}^{C}\left(1-\beta_{s}\right)}}^{+\infty }{\left[\alpha_{f}^{CC}-\frac{\left(Q_{1}+q_{1}\right)w_{f}}{1-\beta_{s}}+c_{f}R_{1}^{C}\right]}^{+}g\left(y\right)dy$. Similar with Proposition 4, we can obtain the following: the optimal threshold of loss is $\alpha_{f}^{CC}=\frac{\left(Q_{1}+q_{1}\right)w_{f}}{1-\beta_{s}}-c_{f}R_{1}^{C}$, and the farmer’s unique optimal $R_{1}^{C*}$ satisfies

$$\begin{array}{c} \int_{0}^{\eta_{1}^{C}}yg(y)dy=\frac{\lambda_{f}c_{f}}{\varphi s_{f}}, \end{array}$$ (7)

where $\eta_{1}^{C}=\frac{Q_{1}^{*}+q_{1}^{*}}{R_{1}^{C*}\left(1-\beta_{s}\right)}$. Thus, the farmer’s optimal expected profit and utility are as follows:

$$\begin{array}{c} E\Pi_{f}^{CC*}=[w_{f}-\varphi s_{f}G\left(\eta_{1}^{C}\right)-\frac{\left(1-\lambda_{f}\right)c_{f}}{\eta_{1}^{C}}]\frac{Q_{1}^{*}+q_{1}^{*}}{1-\beta_{s}},\\ EU_{f}^{CC*}=[w_{f}-\frac{\varphi s_{f}G\left(\eta_{1}^{C}\right)}{\lambda_{f}}]\frac{Q_{1}^{*}+q_{1}^{*}}{1-\beta_{s}}. \end{array}$$ (8)

The supplier’s profit function is $\Pi_{s}^{CC}=o_{1}q_{1}+w_{r}\left(1-\beta_{r}\right)Q_{1}+e\;\text{min}\left[\left(1-\beta_{r}\right)q_{1},{\left[x-\left(1-\beta_{r}\right)Q_{1}\right]}^{+}\right]-\left(1-\phi \right)s_{f}{\left(\frac{Q_{1}+q_{1}}{1-\beta_{s}}-yR_{1}^{C}\right)}^{+}-\frac{\left(Q_{1}+q_{1}\right)w_{f}}{1-\beta_{s}}-c_{s}\left(Q_{1}+q_{1}\right)$. Thus, in the call option contract with a replenishment cost-sharing contract, the supplier’s optimal expected profit is $E\Pi_{s}^{CC*}=e\left[\Gamma \left(Q_{1}^{*}+q_{1}^{*}\right)-\Gamma \left(Q_{1}^{*}\right)\right]+(\frac{p\lambda_{r}o_{1}}{p-e}-\frac{w_{f}+\left(1-\phi \right)s_{f}G\left(\eta_{1}^{C}\right)}{1-\beta_{s}}-c_{s})\left(Q_{1}^{*}+q_{1}^{*}\right)+\left(1-\lambda_{r}\right)\left[\left(w_{r}Q_{1}^{*}+eq_{1}^{*}\right)\left(1-\beta_{r}\right)+o_{1}q_{1}^{*}\right]+\frac{\left(1-\phi \right)\lambda_{f}c_{f}R_{1}^{C*}}{\phi }$.

**Proposition 6** *If* $o_{1}=\frac{\left(p-e\right)[c_{s}+c_{\eta }-p\left(1-\beta_{r}\right)\left(1-\lambda_{r}\right)]}{\lambda_{r}p}$ , *φ* = λ_*f*_, *and* $\frac{w_{r}\left(1-\beta_{r}\right)-o_{1}}{1-\beta_{r}}<e<\frac{p[w_{r}\left(1-\beta_{r}\right)-o_{1}]}{w_{r}\left(1-\beta_{r}\right)}$, *then* $Q_{1}^{*}+q_{1}^{*}=Q_{T}^{*}$ *and* $R_{1}^{C*}=R_{T}^{*}$.

**Fig 2:**
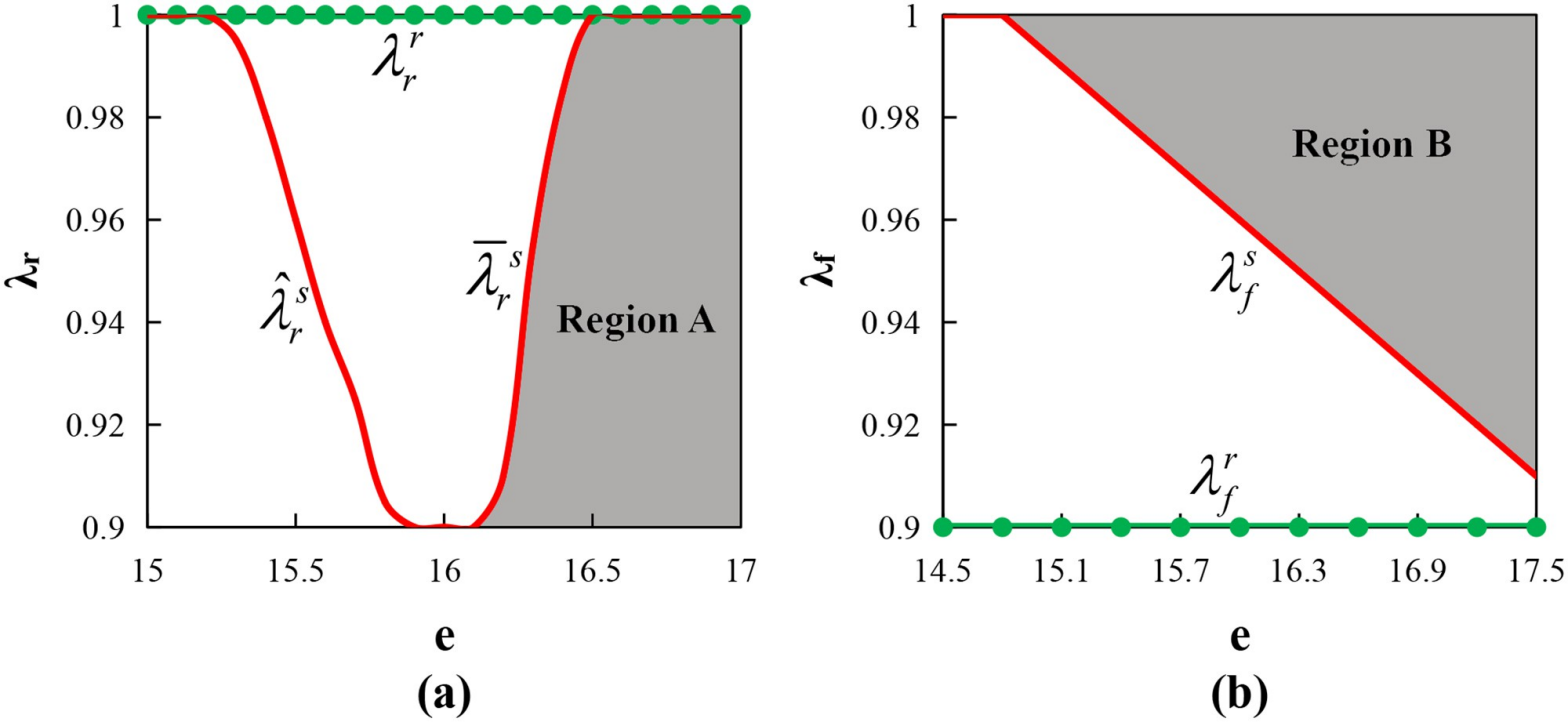
Impact of risk aversion on the Pareto interval.

**Fig 3:**
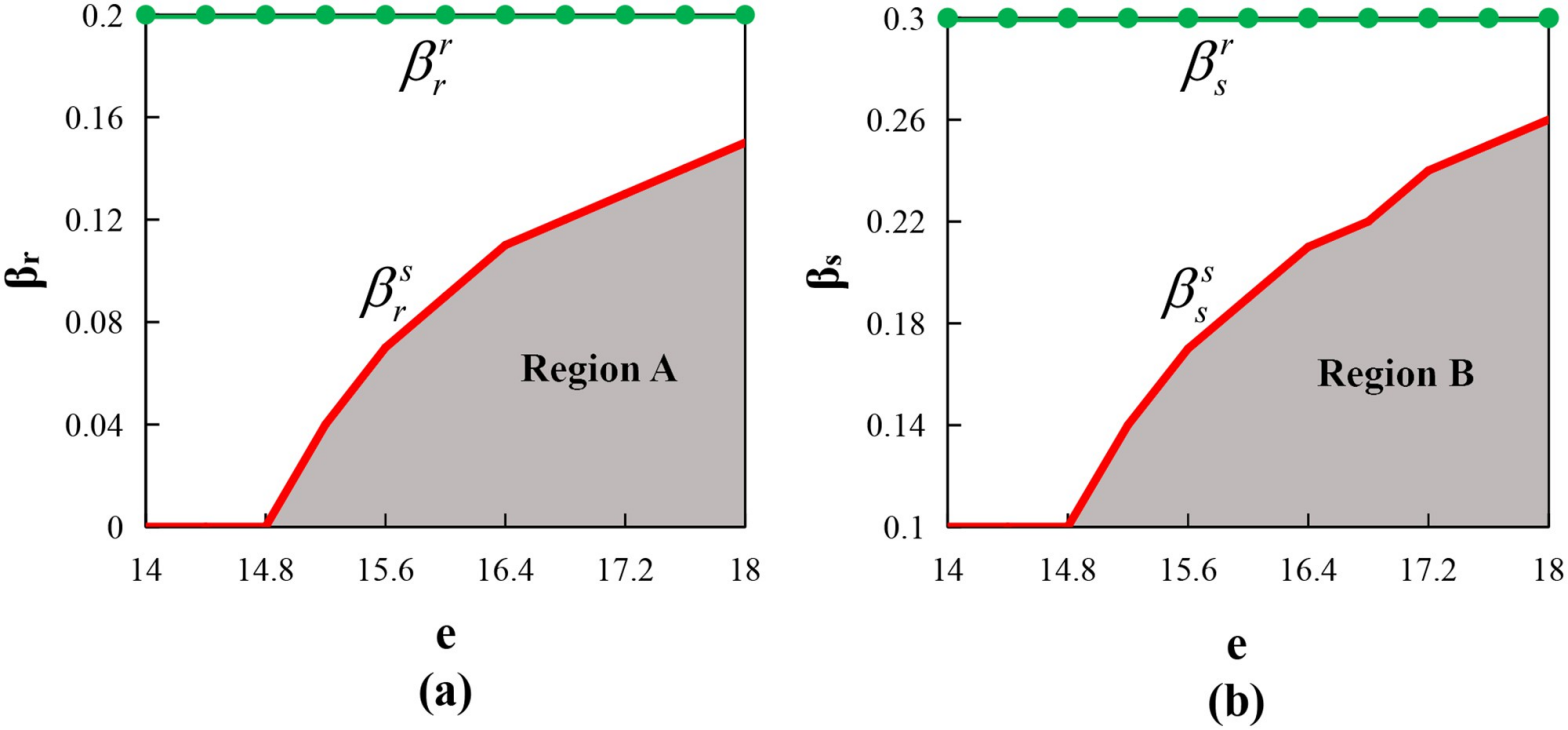
Impact of loss rate on the Pareto interval.

Proposition 6 indicates that under certain conditions, the supply chain can be fully coordinated. Figs 2 and 3 (where *φ* = λ_*f*_ = 0.95, other parameters are the same as above) show that the supply chain can also achieve Pareto improvement under certain conditions. They show that if the supplier wants to coordinate the behavior of the farmer with risk aversion, then he needs to reduce the farmer’s production input and overproduction costs by sharing the farmer’s risk aversion degree, thereby increasing the total profit of the supply chain. Therefore, in essence, the replenishment cost-sharing contract is a type of risk-sharing mechanism. Furthermore, it further illustrates that the supply chain coordination is a process of risk transfer. That is, the supplier first shares the risk of the farmer through setting the replenishment cost sharing ratio *φ*, then transfers the risk to the retailer through setting the call option parameter (*o*_1_, *e*), finally achieving the coordination of the entire supply chain.

In Fig 2(a), when $\lambda_{r}<{\bar{\lambda }}_{r}^{s}$, $E\Pi_{s}^{CC*}>E\Pi_{s}^{DD*}$; when $\lambda_{r}<\lambda_{r}^{r}$, $E\Pi_{r}^{CC*}>E\Pi_{r}^{DD*}$. Therefore, Region A represents the area of Pareto improvement. We determined that the length of the Pareto improvement interval decreases with the retailer’s risk aversion coefficient λ_*r*_. When the retailer’s risk aversion degree is smaller, the supplier can share his risk by lowering the exercise price *e* a little, meaning the supplier can increase *e* appropriately to increase its own profits. In Fig 2(b), when $\lambda_{f}>\lambda_{f}^{s}$, $E\Pi_{s}^{CC*}>E\Pi_{s}^{DD*}$; when $\lambda_{f}>\lambda_{f}^{r}$, $E\Pi_{r}^{CC*}>E\Pi_{r}^{DD*}$. Therefore, Region B represents the area of Pareto improvement. We determined that the length of the Pareto improvement interval increases with the farmer’s risk-averse coefficient. When the farmer’s risk-averse degree is smaller, the supplier will share less replenishment cost (because *φ* = λ_*f*_). Then a smaller *e* can ensure his profit increase, which can provide the retailer with an incentive to increase the order quantity.

In Fig 3(a), when $\beta_{r}<\beta_{r}^{s}$, $E\Pi_{s}^{CC*}>E\Pi_{s}^{DD*}$; when $\beta_{r}<\beta_{r}^{r}$, $E\Pi_{r}^{CC*}>E\Pi_{r}^{DD*}$. Therefore, Region A represents the area of Pareto improvement. We determined that the length of the Pareto improvement interval decreases with the loss rate in distribution process from the supplier to the retailer. In Fig 3(b), when $\beta_{s}<\beta_{s}^{s}$, $E\Pi_{s}^{CC*}>E\Pi_{s}^{DD*}$; when $\beta_{s}<\beta_{s}^{r}$, $E\Pi_{r}^{CC*}>E\Pi_{r}^{DD*}$. Therefore, Region B represents the area of Pareto improvement. We determined that the length of the Pareto improvement interval also decreases with the loss rate in the distribution process from the farmer to the supplier. This shows that the supplier, who is the bearer of the loss, can guarantee his own profit by increasing the call option exercise price when the loss rate is larger; however, when the loss rate is excessively large, the supplier will not be able to compensate for this part of the loss, in which he will not be able to increase his own profit, that is, the supply chain cannot achieve Pareto improvement.

**Corollary 1** *In the call option contract*,

1.
$\frac{\partial Q_{1}^{*}}{\partial \lambda_{r}}>0$ , $\frac{\partial (Q_{1}^{*}+q_{1}^{*})}{\partial \lambda_{r}}>0$, $\frac{\partial R_{1}^{*}}{\partial \lambda_{r}}>0$, $\frac{\partial R_{1}^{C*}}{\partial \lambda_{r}}>0$, $\frac{\partial R_{1}^{*}}{\partial \lambda_{f}}<0$, $\frac{\partial R_{1}^{C*}}{\partial \lambda_{f}}<0$.
2.
$\frac{\partial Q_{1}^{*}}{\partial o_{1}}>0$ , $\frac{\partial (Q_{1}^{*}+q_{1}^{*})}{\partial o_{1}}<0$, $\frac{\partial q_{1}^{*}}{\partial o_{1}}<0$, $\frac{\partial R_{1}^{*}}{\partial o_{1}}<0$, $\frac{\partial R_{1}^{C*}}{\partial o_{1}}<0$, $\frac{\partial Q_{1}^{*}}{\partial e}>0$, $\frac{\partial (Q_{1}^{*}+q_{1}^{*})}{\partial e}<0$, $\frac{\partial q_{1}^{*}}{\partial e}<0$, $\frac{\partial R_{1}^{*}}{\partial e}<0$, $\frac{\partial R_{1}^{C*}}{\partial e}<0$.

Corollary 1(1) shows that the higher the degree of the retailer’s risk aversion, the more conservative his estimate of market demand will be, the smaller his initial order quantity and total order quantity will be, and the farmer’s production input will also decrease. However, the higher the degree of the farmer’s risk aversion, the more he will increase the production input to deal with the uncertain output risks and high replenishment costs. Corollary 1(2) shows that when the option price or exercise price increases, the retailer will increase the initial order quantity and reduce the option order quantity to avoid the high cost of option ordering, and the total order quantity will also become lower. The production input of the farmer would be reduced accordingly.

### 5.2 Put option contracts

#### 5.2.1 Put option contract with wholesale price contract (PO)

In this part, the retailer can execute put options to return the products at a price *e* to the supplier when the actual market demand is lower than the initial quantity *Q*_2_. Thus, the retailer’s profit function is $\Pi_{r}^{PO}=p\;\text{min}\left[\left(1-\beta_{r}\right)Q_{2},x\right]-o_{2}q_{2}-w_{r}\left(1-\beta_{r}\right)Q_{2}+e{\left[\;\text{min}\left[\left(1-\beta_{r}\right)Q_{2}-x,\left(1-\beta_{r}\right)q_{2}\right]\right]}^{+}$. The first term is the retailer’s sales revenue, the second term is the retailer’s option ordering cost, the third term is the retailer’s initial ordering cost, and the fourth term is the revenue of exercising put options. The utility function of the risk-averse retailer is

$$\begin{array}{c} EU_{r}^{PO}=\alpha_{r}^{PO}-\frac{1}{\lambda_{r}}\int_{0}^{\left(1-\beta_{r}\right)\left(Q_{2}-q_{2}\right)}{\left[\alpha_{r}^{PO}-e\left(1-\beta_{r}\right)q_{2}+w_{r}\left(1-\beta_{r}\right)Q_{2}+o_{2}q_{2}-px\right]}^{+}f\left(x\right)dx\\ -\frac{1}{\lambda_{r}}\int_{\left(1-\beta_{r}\right)\left(Q_{2}-q_{2}\right)}^{\left(1-\beta_{r}\right)Q_{2}}{\left[\alpha_{r}^{PO}-\left(e-w_{r}\right)\left(1-\beta_{r}\right)Q_{2}+o_{2}q_{2}-\left(p-e\right)x\right]}^{+}f\left(x\right)dx\\ -\frac{1}{\lambda_{r}}\int_{\left(1-\beta_{r}\right)Q_{2}}^{+\infty }{\left[\alpha_{r}^{PO}-\left(p-w_{r}\right)\left(1-\beta_{r}\right)Q_{2}+o_{2}q_{2}\right]}^{+}f\left(x\right)dx. \end{array}$$

Then, the farmer’s profit function is $\Pi_{f}^{PO}=\frac{Q_{2}w_{f}}{1-\beta_{s}}-c_{f}R_{2}-s_{f}{\left(\frac{Q_{2}}{1-\beta_{s}}-yR_{2}\right)}^{+}$ and utility function of the farmer is $EU_{f}^{PO}=\alpha_{f}^{PO}-\frac{1}{\lambda_{f}}\int_{0}^{\frac{Q_{2}}{R_{2}\left(1-\beta_{s}\right)}}{\left(\alpha_{f}^{PO}-\frac{Q_{2}w_{f}}{1-\beta_{s}}+c_{f}R_{2}+\frac{s_{f}Q_{2}}{1-\beta_{s}}-s_{f}R_{2}y\right)}^{+}g\left(y\right)dy-\frac{1}{\lambda_{f}}\int_{\frac{Q_{2}}{R_{2}\left(1-\beta_{s}\right)}}^{+\infty }{\left(\alpha_{f}^{PO}-\frac{Q_{2}w_{f}}{1-\beta_{s}}+c_{f}R_{2}\right)}^{+}g\left(y\right)dy$. The equilibrium for a decentralized supply chain with the put option contract is described below in Proposition 7.

**Proposition 7** *In the put option contract, suppose* *e*(*p* − *w*_*r*_)(1 − *β*_*r*_)>*po*_2_,

1. *For the retailer, the optimal threshold of loss is* $\alpha_{r}^{PO}=\left(p-w_{r}\right)\left(1-\beta_{r}\right)Q_{2}-o_{2}q_{2}$ . $EU_{r}^{PO}$ *is jointly concave with* *Q*_2_ *and* *q*_2_, *and optimal* $\left(Q_{2}^{*},q_{2}^{*}\right)$ *are uniquely solved by*
  $$\begin{array}{c} Q_{2}^{*}=\frac{1}{1-\beta_{r}}F^{-1}(\frac{\lambda_{r}\left(\left(p-w_{r}\right)\left(1-\beta_{r}\right)-o_{2}\right)}{\left(p-e\right)\left(1-\beta_{r}\right)}),\\ q_{2}^{*}=\frac{1}{1-\beta_{r}}F^{-1}(\frac{\lambda_{r}\left(\left(p-w_{r}\right)\left(1-\beta_{r}\right)-o_{2}\right)}{\left(p-e\right)\left(1-\beta_{r}\right)})-\frac{1}{1-\beta_{r}}F^{-1}(\frac{\lambda_{r}o_{2}}{e(1-\beta_{r})}). \end{array}$$
2. *For the farmer, the optimal threshold of loss is* $\alpha_{f}^{PO}=\frac{Q_{2}w_{f}}{1-\beta_{s}}-c_{f}R_{2}$ , *and the unique optimal* $R_{2}^{*}$ *satisfies*
  $$\begin{array}{c} \int_{0}^{\frac{Q_{2}^{*}}{R_{2}^{*}\left(1-\beta_{s}\right)}}yg(y)dy=\frac{\lambda_{f}c_{f}}{s_{f}}. \end{array}$$ (9)

From Proposition 7(1), $Q_{2}^{*}-q_{2}^{*}<Q_{0}^{*}<Q_{2}^{*}$ and the retailer’s maximum expected profit and utility are $E\Pi_{r}^{PO*}=\left(p-e\right)\Gamma \left(Q_{2}^{*}\right)+e\Gamma \left(Q_{2}^{*}-q_{2}^{*}\right)+\left(1-\lambda_{r}\right)\left[\left(1-\beta_{r}\right)\left(p-w_{r}\right)Q_{2}^{*}-o_{2}q_{2}^{*}\right]$ and $EU_{r}^{PO*}=\frac{p-e}{\lambda_{r}}\Gamma \left(Q_{2}^{*}\right)+\frac{e}{\lambda_{r}}\Gamma \left(Q_{2}^{*}-q_{2}^{*}\right)+\lambda_{r}\left[\left(1-\beta_{r}\right)\left(p-w_{r}\right)Q_{2}^{*}-o_{2}q_{2}^{*}\right]$, respectively. From Eqs (2) and (9), $\eta_{1}=\frac{Q_{0}^{*}}{R_{0}^{*}\left(1-\beta_{s}\right)}=\frac{Q_{2}^{*}}{R_{2}^{*}\left(1-\beta_{s}\right)}$. Thus, the farmer’s optimal expected profit and utility are as follows:

$$\begin{array}{c} E\Pi_{f}^{PO*}=[w_{f}-s_{f}G\left(\eta_{1}\right)-\frac{\left(1-\lambda_{f}\right)c_{f}}{\eta_{1}}]\frac{Q_{2}^{*}}{1-\beta_{s}},\\ EU_{f}^{PO*}=[w_{f}-\frac{s_{f}G\left(\eta_{1}\right)}{\lambda_{f}}]\frac{Q_{2}^{*}}{1-\beta_{s}}. \end{array}$$ (10)

From Eqs (3) and (10), there are $E\Pi_{f}^{PO*}>E\Pi_{f}^{DD*}$ and $EU_{f}^{PO*}>EU_{f}^{DD*}$. It shows that after adding the put option contract, the farmer’s optimal expected profit and utility will be improved. The supplier’s profit function is $\Pi_{s}^{PO}=o_{2}q_{2}+w_{r}\left(1-\beta_{r}\right)Q_{2}-e{\left[\;\text{min}\left[\left(1-\beta_{r}\right)q_{2},\left(1-\beta_{r}\right)Q_{2}-x\right]\right]}^{+}-\frac{Q_{2}w_{f}}{1-\beta_{s}}-c_{s}Q_{2}$. Thus, in the put option contract with the wholesale price contract, the supplier’s optimal expected profit is $E\Pi_{s}^{PO*}=e\left[\Gamma \left(Q_{2}^{*}\right)-\Gamma \left(Q_{2}^{*}-q_{2}^{*}\right)\right]+\left(1-\lambda_{r}\right)o_{2}q_{2}^{*}+[\left(1-\beta_{r}\right)[w_{r}-\frac{e\lambda_{r}\left(p-w_{r}\right)}{p-e}]+\frac{p\lambda_{r}o_{2}}{p-e}-\frac{w_{f}}{1-\beta_{s}}-c_{s}]Q_{2}^{*}$.

**Proposition 8** *In the put option contract*,

1. *If* λ_*f*_ = 1, $o_{2}=\frac{\left(p-e\right)\left(c_{s}+c_{\eta }\right)-p\left(1-\beta_{r}\right)\left[p\left(1-\lambda_{r}\right)+\lambda_{r}w_{r}-e\right]}{\lambda_{r}p}$, *and* $\frac{po_{2}}{\left(p-w_{r}\right)\left(1-\beta_{r}\right)}<e<\frac{w_{r}\left(1-\beta_{r}\right)+o_{2}}{1-\beta_{r}}$, *then* $Q_{2}^{*}=Q_{T}^{*}$ *and* $R_{2}^{*}=R_{T}^{*}$.
2. *If* 0 < λ_*f*_ < 1, *then for any* *o*_2_ *and* *e*, *that is* $\frac{Q_{2}^{*}}{R_{2}^{*}}<\frac{Q_{T}^{*}}{R_{T}^{*}}$.

**Fig 4:**
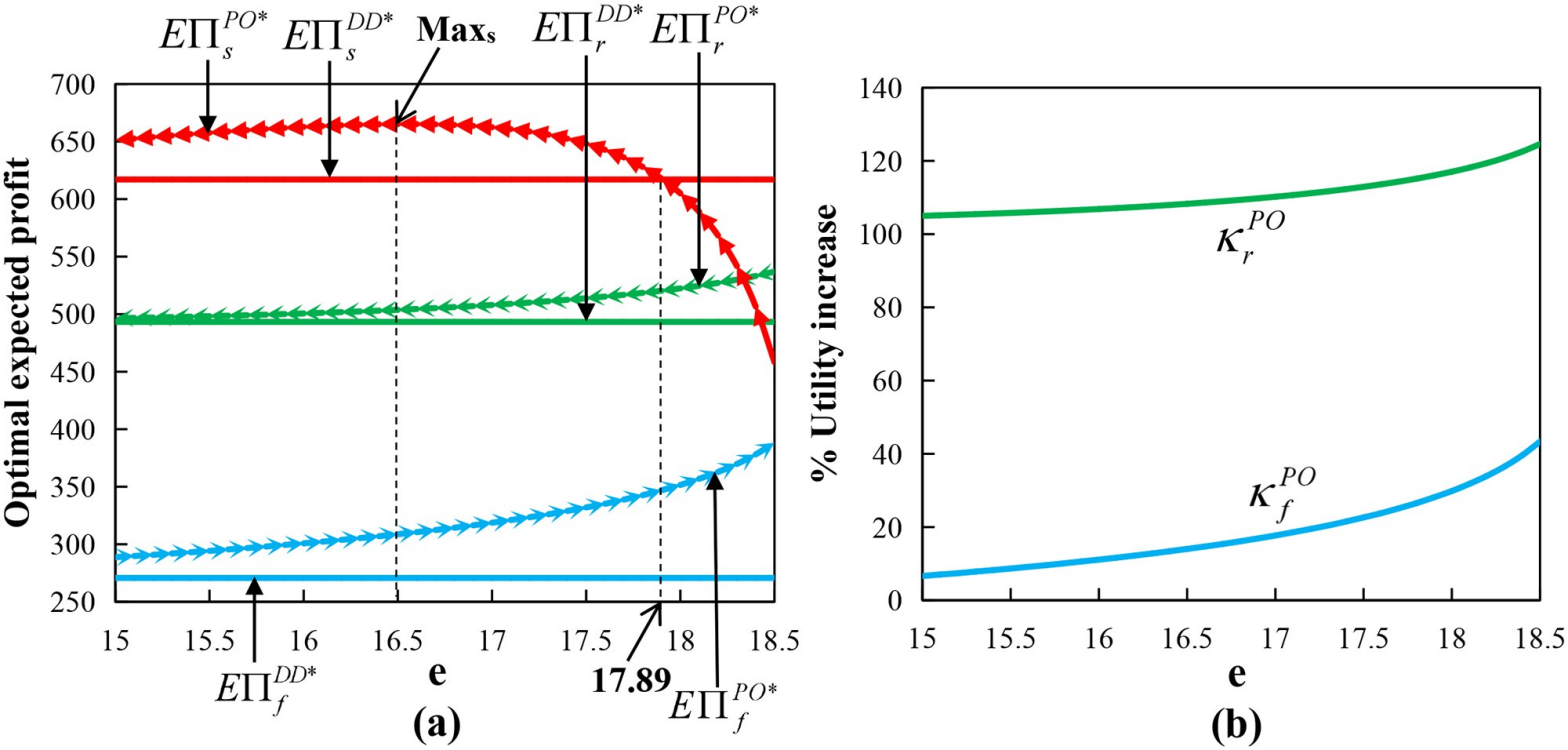
Impact of option exercise price under put option contract with wholesale price contract.

Proposition 8(1) stated that under certain conditions, the supply chain can be fully coordinated. According to Proposition 14 and Fig 1(a), we determined that the supply chain under the put option contract can also achieve Pareto improvement in the interval of (14.79, 17.5), which is similar to that under the call option contract. Proposition 8(2) stated that the order quantity and production input under the put option contract can never be simultaneously equal to those under the centralized decision when the farmer is risk-averse with any *o*_2_ and *e*, that is, the conditions for full coordination cannot be reached. However, Fig 4(a) shows that the supply chain can achieve Pareto improvement in the interval of (15, 17.89). In Fig 4, *o*_2_ = 2 and other parameters are the same as above.

In Fig 4(b), $\kappa_{r}^{PO}=\frac{EU_{r}^{PO*}-EU_{r}^{DD*}}{EU_{r}^{DD*}}*100\%$ represents the retailer’s optimal utility increase ratio and $\kappa_{f}^{PO}=\frac{EU_{f}^{PO*}-EU_{f}^{DD*}}{EU_{f}^{DD*}}*100\%$ represents the farmer’s optimal utility increase ratio in the put option contract. We determined that the optimal utility of both the retailer and the farmer in the put option contract is increased by comparing with decentralized supply chain, especially when the utility of the retailer doubles. Moreover, the increase ratio increases with the put option exercise price. Therefore, the larger exercise price is better for the retailer and farmer when given the option price on profit or utility.

#### 5.2.2 Put option contract with replenishment cost-sharing contract (PC)

Similar to the call option contract, we considered a replenishment cost-sharing contract in the transaction between the supplier and farmer in the following. Thus, we determined that $E\Pi_{r}^{PC*}=E\Pi_{r}^{PO*}$, and the farmer’s profit function is $\Pi_{f}^{PC}=\frac{Q_{2}w_{f}}{1-\beta_{s}}-c_{f}R_{2}^{C}-\phi s_{f}{\left(\frac{Q_{2}}{1-\beta_{s}}-yR_{2}^{C}\right)}^{+}$. Then, his utility function is $EU_{f}^{PC}=\alpha_{f}^{PC}-\frac{1}{\lambda_{f}}\int_{0}^{\frac{Q_{2}}{R_{2}^{C}\left(1-\beta_{s}\right)}}{\left(\alpha_{f}^{PC}-\frac{Q_{2}w_{f}}{1-\beta_{s}}+c_{f}R_{2}^{C}+\frac{\phi s_{f}Q_{2}}{1-\beta_{s}}-\phi s_{f}R_{2}^{C}y\right)}^{+}g\left(y\right)dy-\frac{1}{\lambda_{f}}\int_{\frac{Q_{2}}{R_{2}^{C}\left(1-\beta_{s}\right)}}^{+\infty }{\left(\alpha_{f}^{PC}-\frac{Q_{2}w_{f}}{1-\beta_{s}}+c_{f}R_{2}^{C}\right)}^{+}g\left(y\right)dy$. Similar with Proposition 4, the optimal threshold of loss is $\alpha_{f}^{PC}=\frac{Q_{2}w_{f}}{1-\beta_{s}}-c_{f}R_{2}^{C}$, and the farmer’s unique optimal $R_{2}^{C*}$ satisfies

$$\begin{array}{c} \int_{0}^{\frac{Q_{2}^{*}}{R_{2}^{C*}\left(1-\beta_{s}\right)}}yg(y)dy=\frac{\lambda_{f}c_{f}}{\varphi s_{f}}. \end{array}$$ (11)

From Eqs (7) and (11), $\eta_{1}^{C}=\frac{Q_{1}^{*}+q_{1}^{*}}{R_{1}^{C*}\left(1-\beta_{s}\right)}=\frac{Q_{2}^{*}}{R_{2}^{C*}\left(1-\beta_{s}\right)}$. Thus, the farmer’s optimal expected profit and utility are as follows:

$$\begin{array}{c} E\Pi_{f}^{PC*}=[w_{f}-\varphi s_{f}G\left(\eta_{1}^{C}\right)-\frac{\left(1-\lambda_{f}\right)c_{f}}{\eta_{1}^{C}}]\frac{Q_{2}^{*}}{1-\beta_{s}},\\ EU_{f}^{PC*}=[w_{f}-\frac{\varphi s_{f}G\left(\eta_{1}^{C}\right)}{\lambda_{f}}]\frac{Q_{2}^{*}}{1-\beta_{s}}. \end{array}$$ (12)

The supplier’s profit function is $\Pi_{s}^{PC}=o_{2}q_{2}+w_{r}\left(1-\beta_{r}\right)Q_{2}-e{\left[\;\text{min}\left[\left(1-\beta_{r}\right)q_{2},\left(1-\beta_{r}\right)Q_{2}-x\right]\right]}^{+}-\left(1-\phi \right)s_{f}{\left(\frac{Q_{2}}{1-\beta_{s}}-yR_{2}^{C}\right)}^{+}-\frac{Q_{2}w_{f}}{1-\beta_{s}}-c_{s}Q_{2}$. Thus, in the put option contract with the replenishment cost-sharing contract, the supplier’s optimal expected profit is $E\Pi_{s}^{PC*}=e\left[\Gamma \left(Q_{2}^{*}\right)-\Gamma \left(Q_{2}^{*}-q_{2}^{*}\right)\right]+\left(1-\lambda_{r}\right)o_{2}q_{2}^{*}+\frac{\left(1-\phi \right)\lambda_{f}c_{f}R_{2}^{C*}}{\phi }+[\left(1-\beta_{r}\right)[w_{r}-\frac{e\lambda_{r}\left(p-w_{r}\right)}{p-e}]+\frac{p\lambda_{r}o_{2}}{p-e}-\frac{w_{f}+\left(1-\phi \right)s_{f}G\left(\eta_{1}^{C}\right)}{1-\beta_{s}}-c_{s}]Q_{2}^{*}$.

**Proposition 9** *If* $o_{2}=\frac{\left(p-e\right)\left(c_{s}+c_{\eta }\right)-p\left(1-\beta_{r}\right)\left[p\left(1-\lambda_{r}\right)+\lambda_{r}w_{r}-e\right]}{\lambda_{r}p}$ , *φ* = λ_*f*_, *and* $\frac{po_{2}}{\left(p-w_{r}\right)\left(1-\beta_{r}\right)}<e<\frac{w_{r}\left(1-\beta_{r}\right)+o_{2}}{1-\beta_{r}}$, *then* $Q_{2}^{*}=Q_{T}^{*}$ *and* $R_{2}^{C*}=R_{T}^{*}$.

Similar to the call option contract with the replenishment cost-sharing contract, Proposition 9 indicated that when the farmer’s replenishment cost sharing ratio is equal to his risk-averse coefficient and (*o*_2_, *e*) meets a certain relationship condition, the supply chain can be fully coordinated. At the same time, according to Proposition 14, as the same as in Figs 2 and 3, the supply chain can also achieve Pareto improvement, and the degree of risk aversion of the retailer and farmer has the same effect on the Pareto improvement interval as those under the call option contract, and so are two kinds of loss rate in the distribution process.

**Corollary 2** *In the put option contract*,

1.
$\frac{\partial Q_{2}^{*}}{\partial \lambda_{r}}>0$ , $\frac{\partial (Q_{2}^{*}-q_{2}^{*})}{\partial \lambda_{r}}>0$, $\frac{\partial R_{2}^{*}}{\partial \lambda_{r}}>0$, $\frac{\partial R_{2}^{C*}}{\partial \lambda_{r}}>0$, $\frac{\partial R_{2}^{*}}{\partial \lambda_{f}}<0$, $\frac{\partial R_{2}^{C*}}{\partial \lambda_{f}}<0$.
2.
$\frac{\partial Q_{2}^{*}}{\partial o_{2}}<0$ , $\frac{\partial (Q_{2}^{*}-q_{2}^{*})}{\partial o_{2}}>0$, $\frac{\partial q_{2}^{*}}{\partial o_{2}}<0$, $\frac{\partial R_{2}^{*}}{\partial o_{2}}<0$, $\frac{\partial R_{2}^{C*}}{\partial o_{2}}<0$, $\frac{\partial Q_{2}^{*}}{\partial e}>0$, $\frac{\partial (Q_{2}^{*}-q_{2}^{*})}{\partial e}<0$, $\frac{\partial q_{2}^{*}}{\partial e}>0$, $\frac{\partial R_{2}^{*}}{\partial e}>0$, $\frac{\partial R_{2}^{C*}}{\partial e}>0$.

Corollary 2(1) shows that the higher the degree of retailer’s risk aversion, the more conservative on risk of market demand will be, the smaller the initial order quantity and minimum expected sales quantity will be, and the farmer’s production input will also decrease. However, the higher the degree of the farmer’s risk aversion, the more he will increase the production input to deal with the uncertain output risks and high replenishment costs. Corollary 2(2) shows that when the option price increases, the retailer will decrease the initial order quantity to reduce the remaining product quantity or the use of put options and decrease more option order quantity to avoid the high cost of option ordering. In this case the minimum expected sales quantity will become higher. The production input of the farmer will be decreased according to the initial order quantity. When the exercise price increases, the result is the opposite of the option price increase.

### 5.3 Bidirectional option contracts

#### 5.3.1 Bidirectional option contract with wholesale price contract (BO)

In this part, the retailer can execute bidirectional options to replenish or return products at a price *e* when the actual market demand is higher or lower than the initial order quantity *Q*_3_. In this case, the retailer’s profit function is $\Pi_{r}^{BO}=p\;\text{min}\left[\left(1-\beta_{r}\right)Q_{3},x\right]+e{\left[\;\text{min}\left[\left(1-\beta_{r}\right)Q_{3}-x,\left(1-\beta_{r}\right)q_{3}\right]\right]}^{+}+\left(p-e\right)\;\text{min}\left[{\left[x-\left(1-\beta_{r}\right)Q_{3}\right]}^{+},\left(1-\beta_{r}\right)q_{3}\right]-o_{3}q_{3}-w_{r}\left(1-\beta_{r}\right)Q_{3}$. The first term is the retailer’s sales profit; the second term is the revenue of exercising put options; the third term is the profit of exercising call options; the fourth term is the retailer’s option ordering cost; and the fifth term is the retailer’s initial ordering cost. The utility function of the risk-averse retailer is

$$\begin{array}{c} EU_{r}^{BO}=\alpha_{r}^{BO}-\frac{1}{\lambda_{r}}\int_{0}^{\left(1-\beta_{r}\right)\left(Q_{3}-q_{3}\right)}{\left[\alpha_{r}^{BO}-e\left(1-\beta_{r}\right)q_{3}+w_{r}\left(1-\beta_{r}\right)Q_{3}+o_{3}q_{3}-px\right]}^{+}f\left(x\right)dx\\ -\frac{1}{\lambda_{r}}\int_{\left(1-\beta_{r}\right)\left(Q_{3}-q_{3}\right)}^{\left(1-\beta_{r}\right)\left(Q_{3}+q_{3}\right)}{\left[\alpha_{r}^{BO}-\left(e-w_{r}\right)\left(1-\beta_{r}\right)Q_{3}+o_{3}q_{3}-\left(p-e\right)x\right]}^{+}f\left(x\right)dx\\ -\frac{1}{\lambda_{r}}\int_{\left(1-\beta_{r}\right)\left(Q_{3}+q_{3}\right)}^{+\infty }{\left[\alpha_{r}^{BO}-\left(p-w_{r}\right)\left(1-\beta_{r}\right)Q_{3}-\left(p-e\right)\left(1-\beta_{r}\right)q_{3}+o_{3}q_{3}\right]}^{+}f\left(x\right)dx. \end{array}$$

Then, the farmer’s profit function is $\Pi_{f}^{BO}=\frac{\left(Q_{3}+q_{3}\right)w_{f}}{1-\beta_{s}}-c_{f}R_{3}-s_{f}{\left(\frac{Q_{3}+q_{3}}{1-\beta_{s}}-yR_{3}\right)}^{+}$ and his utility function is $EU_{f}^{BO}=\alpha_{f}^{BO}-\frac{1}{\lambda_{f}}\int_{0}^{\frac{Q_{3}+q_{3}}{R_{3}\left(1-\beta_{s}\right)}}{\left[\alpha_{f}^{BO}-\frac{\left(Q_{3}+q_{3}\right)w_{f}}{1-\beta_{s}}+c_{f}R_{3}+\frac{s_{f}\left(Q_{3}+q_{3}\right)}{1-\beta_{s}}-s_{f}R_{3}y\right]}^{+}g\left(y\right)dy-\frac{1}{\lambda_{f}}\int_{\frac{Q_{3}+q_{3}}{R_{3}\left(1-\beta_{s}\right)}}^{+\infty }{\left[\alpha_{f}^{BO}-\frac{\left(Q_{3}+q_{3}\right)w_{f}}{1-\beta_{s}}+c_{f}R_{3}\right]}^{+}g\left(y\right)dy$. The equilibrium for a decentralized supply chain with the bidirectional option contract is described below in Proposition 10.

**Proposition 10** *In the bidirectional option contract, suppose* (*pw*_*r*_ + *pe* − 2*ew*_*r*_)(1 − *β*_*r*_)>*po*_3_,

1. *For the retailer, the optimal threshold of loss is* $\alpha_{r}^{BO}=\left(p-w_{r}\right)\left(1-\beta_{r}\right)Q_{3}+\left(p-e\right)\left(1-\beta_{r}\right)q_{3}-o_{3}q_{3}$ . $EU_{r}^{BO}$ *is jointly concave with* *Q*_3_ and *q*_3_, *and optimal* $\left(Q_{3}^{*},q_{3}^{*}\right)$ *are uniquely solved by*
  $$\begin{array}{c} Q_{3}^{*}=\frac{1}{2(1-\beta_{r})}[F^{-1}(\frac{\lambda_{r}\left(\left(2p-e-w_{r}\right)\left(1-\beta_{r}\right)-o_{3}\right)}{2\left(p-e\right)\left(1-\beta_{r}\right)})+F^{-1}(\frac{\lambda_{r}\left(\left(e-w_{r}\right)\left(1-\beta_{r}\right)+o_{3}\right)}{2e(1-\beta_{r})})],\\ q_{3}^{*}=\frac{1}{2(1-\beta_{r})}[F^{-1}(\frac{\lambda_{r}\left(\left(2p-e-w_{r}\right)\left(1-\beta_{r}\right)-o_{3}\right)}{2\left(p-e\right)\left(1-\beta_{r}\right)})-F^{-1}(\frac{\lambda_{r}\left(\left(e-w_{r}\right)\left(1-\beta_{r}\right)+o_{3}\right)}{2e(1-\beta_{r})})]. \end{array}$$
2. *For the farmer, the optimal threshold of loss is* $\alpha_{f}^{BO}=\frac{\left(Q_{3}+q_{3}\right)w_{f}}{1-\beta_{s}}-c_{f}R_{3}$ , *and the unique optimal* $R_{3}^{*}$ *satisfies*
  $$\begin{array}{c} \int_{0}^{\frac{Q_{3}^{*}+q_{3}^{*}}{R_{3}^{*}\left(1-\beta_{s}\right)}}yg(y)dy=\frac{\lambda_{f}c_{f}}{s_{f}}. \end{array}$$ (13)

From proposition 10(1), there is $Q_{3}^{*}-q_{3}^{*}<Q_{0}^{*}<Q_{3}^{*}+q_{3}^{*}$. Then, the retailer’s maximum expected profit and utility are $E\Pi_{r}^{BO*}=\left(p-e\right)\Gamma \left(Q_{3}^{*}+q_{3}^{*}\right)+e\Gamma \left(Q_{3}^{*}-q_{3}^{*}\right)+\left(1-\lambda_{r}\right)\left[\left(1-\beta_{r}\right)\left[\left(p-w_{r}\right)Q_{3}^{*}+\left(p-e\right)q_{3}^{*}\right]-o_{3}q_{3}^{*}\right]$ and $EU_{r}^{BO*}=\frac{p-e}{\lambda_{r}}\Gamma \left(Q_{3}^{*}+q_{3}^{*}\right)+\frac{e}{\lambda_{r}}\Gamma \left(Q_{3}^{*}-q_{3}^{*}\right)+\lambda_{r}\left[\left(1-\beta_{r}\right)\left[\left(p-w_{r}\right)Q_{3}^{*}+\left(p-e\right)q_{3}^{*}\right]-o_{3}q_{3}^{*}\right]$, respectively. From Eqs (2) and (13), $\eta_{1}=\frac{Q_{0}^{*}}{R_{0}^{*}\left(1-\beta_{s}\right)}=\frac{Q_{3}^{*}+q_{3}^{*}}{R_{3}^{*}\left(1-\beta_{s}\right)}$. Thus, the farmer’s optimal expected profit and utility are as follows:

$$\begin{array}{c} E\Pi_{f}^{BO*}=[w_{f}-s_{f}G\left(\eta_{1}\right)-\frac{\left(1-\lambda_{f}\right)c_{f}}{\eta_{1}}]\frac{Q_{3}^{*}+q_{3}^{*}}{1-\beta_{s}},\\ EU_{f}^{BO*}=[w_{f}-\frac{s_{f}G\left(\eta_{1}\right)}{\lambda_{f}}]\frac{Q_{3}^{*}+q_{3}^{*}}{1-\beta_{s}}. \end{array}$$ (14)

The Eqs (3) and (14) show that $E\Pi_{f}^{BO*}>E\Pi_{f}^{DD*}$ and $EU_{f}^{BO*}>EU_{f}^{DD*}$, which means that after adding the bidirectional option contract, the farmer’s optimal expected profit and utility will also be improved. The supplier’s profit function is $\Pi_{s}^{BO}=o_{3}q_{3}+w_{r}\left(1-\beta_{r}\right)Q_{3}-\frac{\left(Q_{3}+q_{3}\right)w_{f}}{1-\beta_{s}}-c_{s}\left(Q_{3}+q_{3}\right)+e\;\text{min}\left[{\left[x-\left(1-\beta_{r}\right)Q_{3}\right]}^{+},\left(1-\beta_{r}\right)q_{3}\right]-e{\left[\;\text{min}\left[\left(1-\beta_{r}\right)Q_{3}-x,\left(1-\beta_{r}\right)q_{3}\right]\right]}^{+}$. Thus, in the bidirectional option contract with the wholesale price contract, the supplier’s optimal expected profit is $E\Pi_{s}^{BO*}=e\left[\Gamma \left(Q_{3}^{*}+q_{3}^{*}\right)-\Gamma \left(Q_{3}^{*}-q_{3}^{*}\right)\right]+w_{r}\left(1-\beta_{r}\right)Q_{3}^{*}+\frac{\lambda_{r}}{2}\left[\left(e-w_{r}\right)\left(1-\beta_{r}\right)+o_{3}\right]\left(Q_{3}^{*}-q_{3}^{*}\right)+\left[o_{3}+e\left(1-\beta_{r}\right)\right]q_{3}^{*}-[\frac{\lambda_{r}e\left(\left(2p-e-w_{r}\right)\left(1-\beta_{r}\right)-o_{3}\right)}{2(p-e)}+\frac{w_{f}}{1-\beta_{s}}+c_{s}]\left(Q_{3}^{*}+q_{3}^{*}\right)$.

**Proposition 11** *In the bidirectional option contract, there are*

1. *If* λ_*f*_ = 1, $o_{3}=\left(2p-e-w_{r}\right)\left(1-\beta_{r}\right)+\frac{2\left(p-e\right)[c_{s}+c_{\eta }-p\left(1-\beta_{r}\right)]}{\lambda_{r}p}$, *and* $\frac{po_{3}-pw_{r}\left(1-\beta_{r}\right)}{\left(p-2w_{r}\right)\left(1-\beta_{r}\right)}<e<\;\text{min}[\frac{p\left(1-\beta_{r}\right)-o_{3}}{1-\beta_{r}},\frac{w_{r}\left(1-\beta_{r}\right)+o_{3}}{1-\beta_{r}}]$, *then* $Q_{3}^{*}+q_{3}^{*}=Q_{T}^{*}$ *and* $R_{3}^{*}=R_{T}^{*}$.
2. *If* 0 < λ_*f*_ < 1, *then for any* *o*_3_ and *e*, *that is* $\frac{Q_{3}^{*}+q_{3}^{*}}{R_{3}^{*}}<\frac{Q_{T}^{*}}{R_{T}^{*}}$.

**Fig 5:**
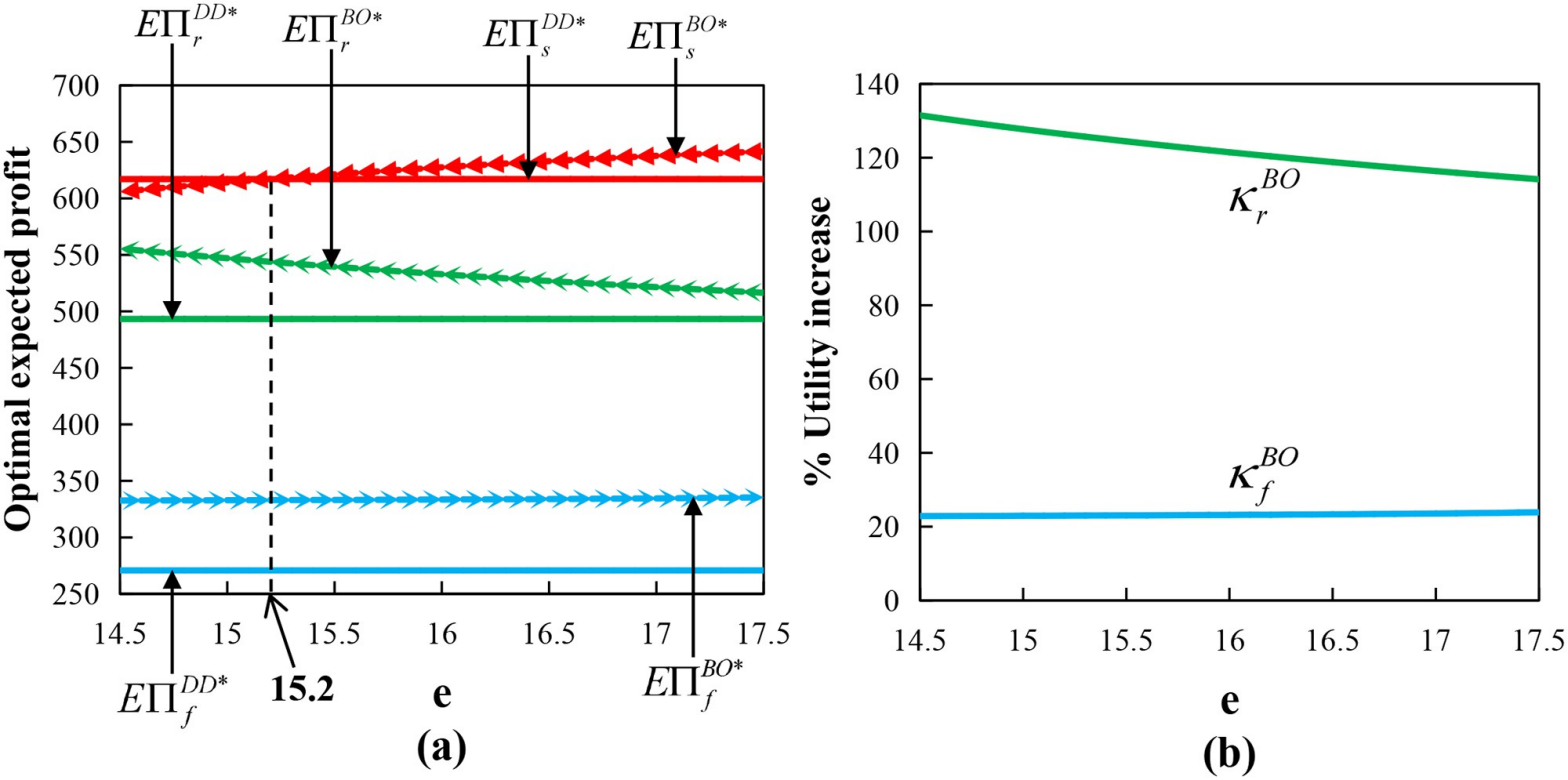
Impact of option exercise price under bidirectional option with wholesale price contract.

Proposition 11(1) indicated that under certain conditions, the supply chain can be fully coordinated. According to Proposition 14 and Fig 1(a), we determined that the supply chain can also achieve Pareto improvement in the interval of (14.79, 17.5), which is similar to that under the call option contract. Proposition 11(2) shows that the order quantity and production input under the bidirectional option contract can never be simultaneously equal to those under the centralized decision when the farmer is risk-averse, that is, the conditions for full coordination cannot be reached. However, Fig 5(a) shows that the supply chain can achieve Pareto improvement in the interval of (15.2, 17.5). In Fig 5, *o*_3_ = 3 and other parameters are the same as above.

In Fig 5(b), $\kappa_{r}^{BO}=\frac{EU_{r}^{BO*}-EU_{r}^{DD*}}{EU_{r}^{DD*}}*100\%$ represents the retailer’s optimal utility increase ratio and $\kappa_{f}^{BO}=\frac{EU_{f}^{BO*}-EU_{f}^{DD*}}{EU_{f}^{DD*}}*100\%$ represents the farmer’s optimal utility increase ratio in the bidirectional option contract. We determined that the optimal utility of both the retailer and the farmer in the bidirectional option contract is increased compared with that in the decentralized decision, with the utility of the retailer more than doubling. However, the retailer’s increase ratio decreases with the bidirectional option exercise price and the farmer’s increase ratio slightly increases. Therefore, the increasing trend of optimal utility and optimal expected profit is consistent.

#### 5.3.2 Bidirectional option contract with replenishment cost-sharing contract (BC)

Similar to the call option contract, we considered a replenishment cost-sharing contract in the transaction between the supplier and farmer. Thus, we determined that $E\Pi_{r}^{BC*}=E\Pi_{r}^{BO*}$, and the farmer’s profit function is $\Pi_{f}^{BC}=\frac{\left(Q_{3}+q_{3}\right)w_{f}}{1-\beta_{s}}-c_{f}R_{3}^{C}-\phi s_{f}{\left(\frac{Q_{3}+q_{3}}{1-\beta_{s}}-yR_{3}^{C}\right)}^{+}$. Then, his utility function is $EU_{f}^{BC}=\alpha_{f}^{BC}-\frac{1}{\lambda_{f}}\int_{0}^{\frac{Q_{3}+q_{3}}{R_{3}^{C}\left(1-\beta_{s}\right)}}{\left[\alpha_{f}^{BC}-\frac{\left(Q_{3}+q_{3}\right)w_{f}}{1-\beta_{s}}+c_{f}R_{3}^{C}+\frac{\phi s_{f}\left(Q_{3}+q_{3}\right)}{1-\beta_{s}}-\phi s_{f}R_{3}^{C}y\right]}^{+}g\left(y\right)dy-\frac{1}{\lambda_{f}}\int_{\frac{Q_{3}+q_{3}}{R_{3}^{C}\left(1-\beta_{s}\right)}}^{+\infty }{\left[\alpha_{f}^{BC}-\frac{\left(Q_{3}+q_{3}\right)w_{f}}{1-\beta_{s}}+c_{f}R_{3}^{C}\right]}^{+}g\left(y\right)dy$. Similar to Proposition 4, we can obtain the optimal threshold of loss is $\alpha_{f}^{BC}=\frac{\left(Q_{3}+q_{3}\right)w_{f}}{1-\beta_{s}}-c_{f}R_{3}^{C}$, and the farmer’s unique optimal $R_{3}^{C*}$ satisfies

$$\begin{array}{c} \int_{0}^{\frac{Q_{3}^{*}+q_{3}^{*}}{R_{3}^{C*}\left(1-\beta_{s}\right)}}yg(y)dy=\frac{\lambda_{f}c_{f}}{\varphi s_{f}}. \end{array}$$ (15)

From Eqs (7) and (15), $\eta_{1}^{C}=\frac{Q_{1}^{*}+q_{1}^{*}}{R_{1}^{C*}\left(1-\beta_{s}\right)}=\frac{Q_{3}^{*}+q_{3}^{*}}{R_{3}^{C*}\left(1-\beta_{s}\right)}$. Thus, the farmer’s optimal expected profit and utility are as follows:

$$\begin{array}{c} E\Pi_{f}^{BC*}=[w_{f}-\varphi s_{f}G\left(\eta_{1}^{C}\right)-\frac{\left(1-\lambda_{f}\right)c_{f}}{\eta_{1}^{C}}]\frac{Q_{3}^{*}+q_{3}^{*}}{1-\beta_{s}},\\ EU_{f}^{BC*}=[w_{f}-\frac{\varphi s_{f}G\left(\eta_{1}^{C}\right)}{\lambda_{f}}]\frac{Q_{3}^{*}+q_{3}^{*}}{1-\beta_{s}}. \end{array}$$ (16)

The supplier’s profit function is $\Pi_{s}^{BC}=o_{3}q_{3}+w_{r}\left(1-\beta_{r}\right)Q_{3}-\frac{\left(Q_{3}+q_{3}\right)w_{f}}{1-\beta_{s}}-c_{s}\left(Q_{3}+q_{3}\right)-\left(1-\phi \right)s_{f}{\left(\frac{Q_{3}+q_{3}}{1-\beta_{s}}-yR_{3}^{C}\right)}^{+}+e\;\text{min}\left[{\left[x-\left(1-\beta_{r}\right)Q_{3}\right]}^{+},\left(1-\beta_{r}\right)q_{3}\right]-e{\left[\;\text{min}\left[\left(1-\beta_{r}\right)Q_{3}-x,\left(1-\beta_{r}\right)q_{3}\right]\right]}^{+}$. Thus, in the bidirectional option contract with the replenishment cost-sharing contract, the supplier’s optimal expected profit is $E\Pi_{s}^{BC*}=e\left[\Gamma \left(Q_{3}^{*}+q_{3}^{*}\right)-\Gamma \left(Q_{3}^{*}-q_{3}^{*}\right)\right]+\left[o_{3}+e\left(1-\beta_{r}\right)\right]q_{3}^{*}+w_{r}\left(1-\beta_{r}\right)Q_{3}^{*}+\frac{\lambda_{r}}{2}\left[\left(e-w_{r}\right)\left(1-\beta_{r}\right)+o_{3}\right]\left(Q_{3}^{*}-q_{3}^{*}\right)+\frac{\left(1-\phi \right)\lambda_{f}c_{f}R_{3}^{C*}}{\phi }-[\frac{\lambda_{r}e\left(\left(2p-e-w_{r}\right)\left(1-\beta_{r}\right)-o_{3}\right)}{2(p-e)}+\frac{w_{f}+\left(1-\phi \right)s_{f}G\left(\eta_{1}^{C}\right)}{1-\beta_{s}}+c_{s}]\left(Q_{3}^{*}+q_{3}^{*}\right)$.

**Proposition 12** *If* $o_{3}=\left(2p-e-w_{r}\right)\left(1-\beta_{r}\right)+\frac{2\left(p-e\right)[c_{s}+c_{\eta }-p\left(1-\beta_{r}\right)]}{\lambda_{r}p}$ , *φ* = λ_*f*_, *and* $\frac{po_{3}-pw_{r}\left(1-\beta_{r}\right)}{\left(p-2w_{r}\right)\left(1-\beta_{r}\right)}<e<\;\text{min}\left[\frac{p\left(1-\beta_{r}\right)-o_{3}}{1-\beta_{r}},\frac{w_{r}\left(1-\beta_{r}\right)+o_{3}}{1-\beta_{r}}\right]$, *then* $Q_{3}^{*}+q_{3}^{*}=Q_{T}^{*}$ *and* $R_{3}^{C*}=R_{T}^{*}$.

Similar to the call option contract with the replenishment cost-sharing contract, Proposition 12 indicated that when the farmer’s replenishment cost sharing ratio is equal to his risk-averse coefficient and (*o*_3_, *e*) meets a certain relationship condition, the supply chain can be fully coordinated. At the same time, according to Proposition 14, and the same as Figs 2 and 3, the supply chain can also achieve Pareto improvement. Moreover, the degree of risk aversion of the retailer and farmer has the same effect on the Pareto improvement interval as those under the call option contract, and so are the two types of loss rates in the distribution process.

**Corollary 3** *In the bidirectional option contract*,

1.
$\frac{\partial Q_{3}^{*}}{\partial \lambda_{r}}>0$ , $\frac{\partial (Q_{3}^{*}+q_{3}^{*})}{\partial \lambda_{r}}>0$, $\frac{\partial (Q_{3}^{*}-q_{3}^{*})}{\partial \lambda_{r}}>0$, $\frac{\partial R_{3}^{*}}{\partial \lambda_{r}}>0$, $\frac{\partial R_{3}^{C*}}{\partial \lambda_{r}}>0$, $\frac{\partial R_{3}^{*}}{\partial \lambda_{f}}<0$, $\frac{\partial R_{3}^{C*}}{\partial \lambda_{f}}<0$.
2.
$\frac{\partial (Q_{3}^{*}+q_{3}^{*})}{\partial o_{3}}<0$ , $\frac{\partial (Q_{3}^{*}-q_{3}^{*})}{\partial o_{3}}>0$, $\frac{\partial q_{3}^{*}}{\partial o_{3}}<0$, $\frac{\partial R_{3}^{*}}{\partial o_{3}}<0$, $\frac{\partial R_{3}^{C*}}{\partial o_{3}}<0$. $\frac{\partial (Q_{3}^{*}-q_{3}^{*})}{\partial e}>0$. *If* *o*_3_ < (*p* − *w*_*r*_)(1 − *β*_*r*_), *then* $\frac{\partial (Q_{3}^{*}+q_{3}^{*})}{\partial e}>0$, $\frac{\partial Q_{3}^{*}}{\partial e}>0$, $\frac{\partial R_{3}^{*}}{\partial e}>0$, *and* $\frac{\partial R_{3}^{C*}}{\partial e}>0$; *if* *o*_3_ > (*p* − *w*_*r*_)(1 − *β*_*r*_), *then* $\frac{\partial (Q_{3}^{*}+q_{3}^{*})}{\partial e}<0$, $\frac{\partial q_{3}^{*}}{\partial e}<0$, $\frac{\partial R_{3}^{*}}{\partial e}<0$, *and* $\frac{\partial R_{3}^{C*}}{\partial e}<0$.

Corollary 3(1) shows that the higher the degree of retailer’s risk aversion, the smaller his initial order quantity, total order quantity, and minimum expected sales quantity will be, and the farmer’s production input will also decrease. However, when the degree of farmer’s risk aversion is higher, the farmer will increase the production input to deal with the uncertain output risks and high replenishment costs. Corollary 3(2) shows that when the option price increases, the retailer will decrease the option order quantity to avoid the high cost of option orders and the total order quantity will be lower. However, the minimum expected sales quantity will become higher. The production input of the farmer will be decreased according to the total order quantity. When the option price is small and the exercise price increases, the retailer will increase the initial order quantity to avoid the high cost of option orders and the total order quantity will be increased. The production input of the farmer will be increased according to the total order quantity. When the option price is high and the exercise price increases, the retailer will decrease the option order quantity to avoid the high cost of option orders and the total order quantity will be decreased. The production input of the farmer will be decreased according to the total order quantity. Finally, the minimum expected sales quantity will be increased by the increase in initial order quantity or the decrease in option order quantity when the exercise price increases.

## 6 Contracts comparison

In this section, we will compare the retailer’s optimal order quantity, the farmer’s optimal production input quantity, and the supply chain members’ optimal expected profits and utilities under three types of option contracts with and without the replenishment cost-sharing contract. Moreover, we will analyze the preferences of supply chain members for these contracts. In particular, we consider the contract selection of the supplier due to the fact that he is the supply chain leader.

**Proposition 13** *When* $w_{r}-\frac{o_{1}}{1-\beta_{r}}<e<\;\text{min}\left(p-\frac{o_{3}}{1-\beta_{r}},w_{r}+\frac{o_{2}}{1-\beta_{r}}\right)$ , *we obtain relations of order quantities and production inputs, which are showed in* Table 1.

**Table 1:** Relations of order quantities and production inputs.

Option prices	Condition of option prices	Relations of order quantities	Relations of production inputs
*o*_1_	$\left(o_{2}-\left(e-w_{r}\right)\left(1-\beta_{r}\right),\frac{o_{3}-\left(e-w_{r}\right)\left(1-\beta_{r}\right)}{2}\right)$	$Q_{2}^{*}>Q_{1}^{*}+q_{1}^{*}>Q_{3}^{*}+q_{3}^{*}$	$R_{2}^{*}>R_{1}^{*}>R_{3}^{*}$ $R_{2}^{C*}>R_{1}^{C*}>R_{3}^{C*}$
	$\left(\frac{o_{3}-\left(e-w_{r}\right)\left(1-\beta_{r}\right)}{2},o_{2}-\left(e-w_{r}\right)\left(1-\beta_{r}\right)\right)$	$Q_{3}^{*}+q_{3}^{*}>Q_{1}^{*}+q_{1}^{*}>Q_{2}^{*}$	$R_{3}^{*}>R_{1}^{*}>R_{2}^{*}$ $R_{3}^{C*}>R_{1}^{C*}>R_{2}^{C*}$
*o*_2_	$\left(o_{1}+\left(e-w_{r}\right)\left(1-\beta_{r}\right),\frac{o_{3}+\left(e-w_{r}\right)\left(1-\beta_{r}\right)}{2}\right)$	$Q_{1}^{*}+q_{1}^{*}>Q_{2}^{*}>Q_{3}^{*}+q_{3}^{*}$	$R_{1}^{*}>R_{2}^{*}>R_{3}^{*}$ $R_{1}^{C*}>R_{2}^{C*}>R_{3}^{C*}$
	$\left(\frac{o_{3}+\left(e-w_{r}\right)\left(1-\beta_{r}\right)}{2},o_{1}+\left(e-w_{r}\right)\left(1-\beta_{r}\right)\right)$	$Q_{3}^{*}+q_{3}^{*}>Q_{2}^{*}>Q_{1}^{*}+q_{1}^{*}$	$R_{3}^{*}>R_{2}^{*}>R_{1}^{*}$ $R_{3}^{C*}>R_{2}^{C*}>R_{1}^{C*}$
*o*_3_	(2*o*_1_ + (*e* − *w*_*r*_)(1 − *β*_*r*_), 2*o*_2_ − (*e* − *w*_*r*_)(1 − *β*_*r*_))	$Q_{1}^{*}+q_{1}^{*}>Q_{3}^{*}+q_{3}^{*}>Q_{2}^{*}$	$R_{1}^{*}>R_{3}^{*}>R_{2}^{*}$ $R_{1}^{C*}>R_{3}^{C*}>R_{2}^{C*}$
	(2*o*_2_ − (*e* − *w*_*r*_)(1 − *β*_*r*_), 2*o*_1_ + (*e* − *w*_*r*_)(1 − *β*_*r*_))	$Q_{2}^{*}>Q_{3}^{*}+q_{3}^{*}>Q_{1}^{*}+q_{1}^{*}$	$R_{2}^{*}>R_{3}^{*}>R_{1}^{*}$ $R_{2}^{C*}>R_{3}^{C*}>R_{1}^{C*}$

**Proposition 14** *If* *o*_2_ = (*e* − *w*_*r*_)(1 − *β*_*r*_) + *o*_1_ *and* *o*_3_ = (*e* − *w*_*r*_)(1 − *β*_*r*_) + 2*o*_1_, *then*

1.
$Q_{1}^{*}+q_{1}^{*}=Q_{2}^{*}=Q_{3}^{*}+q_{3}^{*}$ , $q_{1}^{*}=q_{2}^{*}=2q_{3}^{*}$, $R_{1}^{*}=R_{2}^{*}=R_{3}^{*}$, $R_{1}^{C*}=R_{2}^{C*}=R_{3}^{C*}$.
2.
$E\Pi_{r}^{CO*}=E\Pi_{r}^{PO*}=E\Pi_{r}^{BO*}$ , $E\Pi_{r}^{CC*}=E\Pi_{r}^{PC*}=E\Pi_{r}^{BC*}$, *and* $EU_{r}^{CO*}=EU_{r}^{PO*}=EU_{r}^{BO*}$.
3.
$E\Pi_{f}^{CO*}=E\Pi_{f}^{PO*}=E\Pi_{f}^{BO*}$ , $E\Pi_{f}^{CC*}=E\Pi_{f}^{PC*}=E\Pi_{f}^{BC*}$, $EU_{f}^{CO*}=EU_{f}^{PO*}=EU_{f}^{BO*}$, *and* $EU_{f}^{CC*}=EU_{f}^{PC*}=EU_{f}^{BC*}$.
4.
$E\Pi_{s}^{CO*}=E\Pi_{s}^{PO*}=E\Pi_{s}^{BO*}$ *and* $E\Pi_{s}^{CC*}=E\Pi_{s}^{PC*}=E\Pi_{s}^{BC*}$.

Proposition 14 indicates that when *o*_1_, *o*_2_, and *o*_3_ meets a certain relationship condition, for the call, put, and bidirectional option contracts, their total order quantities are equal and their production inputs are also equal. According to Proposition 14(2)(3)(4), the profits and utilities of supply chain members are unchanged under the three option contracts, which is the same for the retailer, supplier, farmer, and the entire supply chain to choose any option contract when *o*_2_ = (*e* − *w*_*r*_)(1 − *β*_*r*_) + *o*_1_ and *o*_3_ = (*e* − *w*_*r*_)(1 − *β*_*r*_) + 2*o*_1_. Therefore, from the view of profit and utility optimization, the supply chain members may select any option contracts that are exactly the same when the supply chain is fully coordinated.

**Fig 6:**
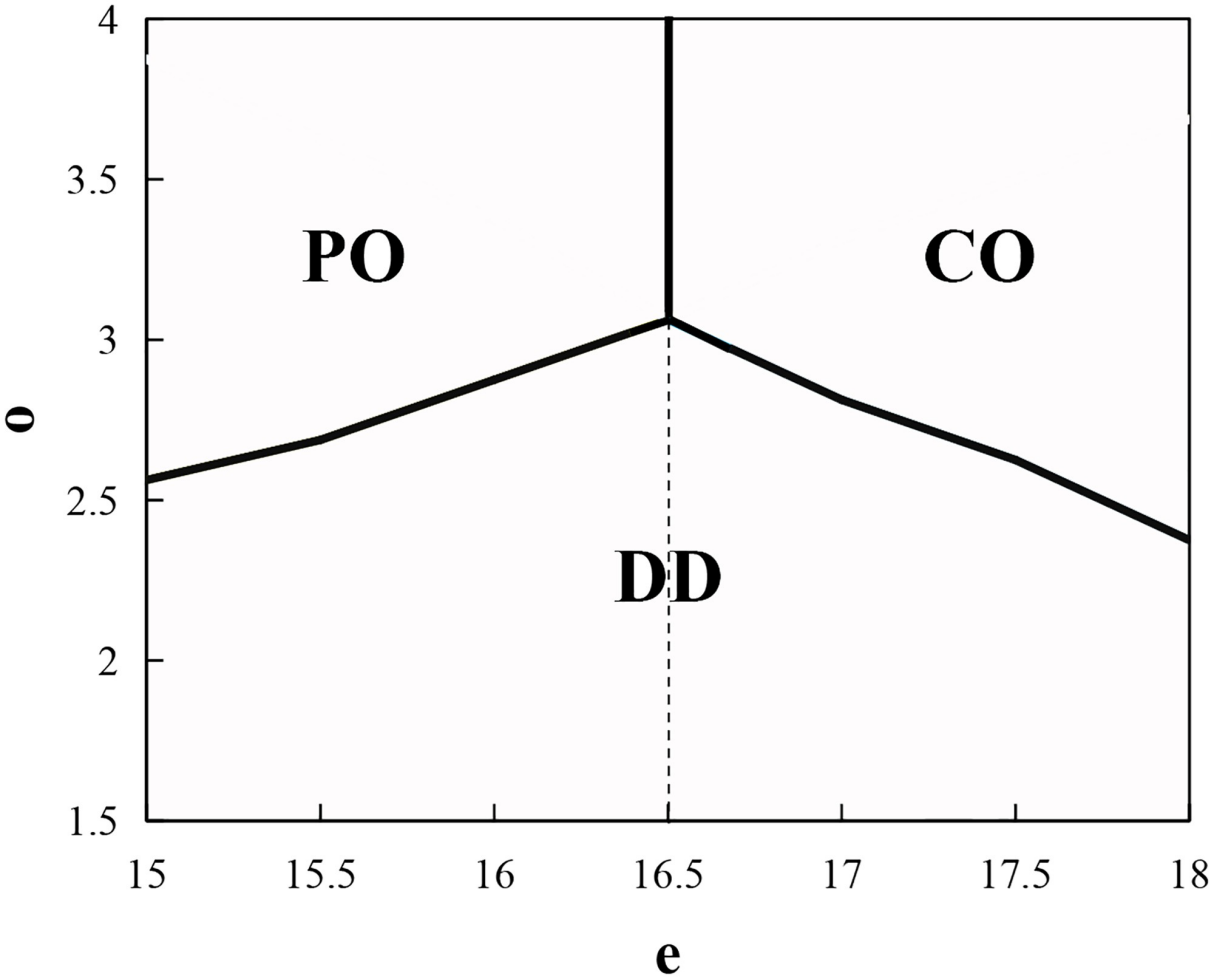
The supplier’s selection of contracts under risk-averse retailer and farmer.

Fig 6 shows the preference of contracts without the replenishment cost-sharing contract for the supplier when the retailer and the farmer are risk-averse, where *o*_3_ = 1.5*o*, *p* = 25, and other parameters are the same as above. As the leader of the supply chain and the designer of contracts, the supplier’s choice of contracts will be the actual choice of the entire supply chain. In Fig 6, all contractual conditions and assumptions are fulfilled. We determined that the supplier will choose the put option contract when *e* is low and *o* is high; he will choose the call option contract when *e* and *o* are high; and he will choose the decentralized decision (wholesale-price-only contract) when *e* is moderate. As the option price *o* decreases, he is more inclined to choose the decentralized decision and generally could not choose a bidirectional option contract.

**Proposition 15** *When the supply chain can be fully coordinated*,

1. *If* $s_{f}>\frac{c_{f}}{\eta G(\eta )}$ , *then: (i)* $E\Pi_{s}^{CO*}>E\Pi_{s}^{CC*}$, $E\Pi_{s}^{PO*}>E\Pi_{s}^{PC*}$, *and* $E\Pi_{s}^{BO*}>E\Pi_{s}^{BC*}$; *(ii)* $E\Pi_{f}^{CO*}<E\Pi_{f}^{CC*}$, $E\Pi_{f}^{PO*}<E\Pi_{f}^{PC*}$, *and* $E\Pi_{f}^{BO*}<E\Pi_{f}^{BC*}$.
2. *If* $c_{f}<s_{f}<\frac{c_{f}}{\eta G(\eta )}$ , *then: (i)* $E\Pi_{s}^{CO*}<E\Pi_{s}^{CC*}$, $E\Pi_{s}^{PO*}<E\Pi_{s}^{PC*}$, *and* $E\Pi_{s}^{BO*}<E\Pi_{s}^{BC*}$; *(ii)* $E\Pi_{f}^{CO*}>E\Pi_{f}^{CC*}$, $E\Pi_{f}^{PO*}>E\Pi_{f}^{PC*}$, *and* $E\Pi_{f}^{BO*}>E\Pi_{f}^{BC*}$.

Under three types of option contracts, Proposition 15 indicated that when the supply chain achieved full coordination, if the farmer’s spot price *s*_*f*_ is greater than $\frac{c_{f}}{\eta G(\eta )}$, then the supplier’s profit under a replenishment cost-sharing contract will be less than the profit without a replenishment cost-sharing contract, indicating that the cost shared by the supplier is higher than the profit brought by the increase in the order quantity. In this situation the supplier sacrifices his own profit to promote the full coordination of the supply chain. The opposite holds for the farmer. When no replenishment cost-sharing contract exists, the farmer bears the high replenishment cost alone. When there is a replenishment cost-sharing contract, the supplier shares part of his high replenishment cost and he will reduce the production cost of the product. Therefore, the farmer’s profit under a replenishment cost-sharing contract will be more than the profit without a replenishment cost-sharing contract. If the farmer’s spot price *s*_*f*_ is less than $\frac{c_{f}}{\eta G(\eta )}$, then the supplier’s profit under a replenishment cost-sharing contract will be more than the profit without one because the cost shared by the supplier is lower than the profit brought by the increase in the order quantity. However, the farmer’s profit under a replenishment cost-sharing contract will be less than the profit without a replenishment cost-sharing contract.

## 7 Conclusions

To improve the balance of supply and demand and reduce profit losses in the contract farming supply chain, upstream and downstream members would jointly increase the profits of each member and the total supply chain by adopting various cooperation mechanisms. Based on extant literature, we used the CVaR criterion to resolve the optimal decisions of risk-averse retailer and farmer. The option contract is implemented between the supplier and the retailer, and the wholesale price contract or the replenishment cost-sharing contract is implemented between the supplier and the farmer. We first established a centralized decision and decentralized decision model to provide the basis of comparison for the full coordination and Pareto improvement. The retailer decides the initial order quantity and option order quantity; the farmer decides the production input quantity; and the supplier, as the leader, sets reasonable contract parameters to obtain full coordination or Pareto improvement. Then, we compared the equilibrium decisions, profits, and utilities of all parties with the basic model and obtained the conditions of full coordination and the area of Pareto improvement. Finally, by comparing the equilibrium decision and profit under various scenarios, we obtained the selection preference of the supply chain members for the contracts. We summarized key findings corresponding to the answers to the research questions posed as follows.

First, when the farmer is risk-neutral, the option contracts with the wholesale price contract can simultaneously achieve the full coordination and Pareto improvement of the supply chain under certain conditions. When the farmer is risk-averse, option contracts with the wholesale price contract can only make each member better off and cannot maximize the supply chain’s profit. However, the option contracts with the replenishment cost-sharing contract can make the risk-averse farmer, the leader supplier, and the risk-averse retailer better off and maximize the total profit of supply chain. Second, the retailer’s optimal total order quantity increases with his risk aversion coefficient and decreases with option price. The farmer’s optimal production input quantity decreases with his risk aversion coefficient and option price and increases with the retailer’s risk aversion coefficient. Moreover, the optimal production input quantity decreases with the option exercise price in the call option contract, increases with the exercise price in the put option contract, and increases first, then decreases with the exercise price in the bidirectional option contract. Third, in the option contracts with a replenishment cost sharing contract, the length of the supply chain’s Pareto improvement interval will decrease with the retailer’s risk aversion coefficient, increase with the farmer’s risk aversion coefficient, and decrease with the loss rate, and the contracts are no longer applicable when the loss rate is excessively large. When the supply chain can achieve full coordination, the profits of the supply chain members will not change in the three option contracts and setting reasonable option parameters will enable them get the same profit. Under the option contracts with a wholesale price contract when both the farmer and the retailer are risk-averse, the leader supplier will not choose a bidirectional option contract in the interval where three option contracts are established.

Our study has important theoretical and practical implications. Academically, under contract farming, this paper investigated whether the three option contracts can encourage the farmer to increase the production input quantity and the retailer to increase order quantity, and can promote the balance of supply and demand, in which the three-level FAP supply chain can be fully coordinated and the profits of supply chain members can increase when the farmer and the retailer are risk-averse. To the best of our knowledge, this paper took the first attempt to simultaneously consider the risk preference of the farmer and the retailer in the three-level FAP supply chain. We incorporated uncertain output and demand, decision-making by the risk-averse farmer and retailer, and three option coordination contracts in a three-level FAP supply chain, which is our main contribution in modeling. In this way, we have enriched the research on the agricultural supply chain and made preliminary explorations to coordinate the supply chain.

In practice, the contract mechanism can not only strengthen the cooperation between the upstream and downstream of the supply chain, but also effectively improve the performance of the supply chain. From our results, we can obtain the following important managerial implications. First, when the farmer is risk-neutral and the retailer is risk-averse, the supplier can provide the retailer with any option contract and the farmer with wholesale price contracts to maximize the total profit of the supply chain and increase the profits of all parties. When the farmer and the retailer are risk-averse, the original contracts can only increase the profits of all parties without maximizing the total profit. Only the supplier shares a part of the farmer’s replenishment costs to maximize the total profit of the supply chain. Moreover, when the three options’ exercise prices satisfied a certain relationship, any option contract provided by the supplier to distribute profits is the same. Second, the degree of risk aversion of the farmer and the retailer will affect the amount of production input and product orders, but option contracts with a replenishment cost-sharing contract can hedge their effect and by designing reasonable contract parameters, the supply chain and its members can be better off. And so is the loss rate in the distribution process. However, when the loss rate is excessively large, these contracts will no longer be used, they will not be beneficial to the leader. Third, it is generally believed that the bidirectional option contract can cope with demand risks better and would benefit all parties in the supply chain. However, our analysis shows that for the dominant supplier, the bidirectional option contract functions poorer than the decentralized decision and the supplier will not provide the retailer with a bidirectional option contract when three option contracts are established and the farmer is risk-averse. In a word, the leader of the supply chain should be aware of the risk aversion degree of all parties and the quantity loss rate of FAPs, then choose the appropriate contract and set reasonable contract parameters.

Although these findings and managerial insights are important, there are still some limitations and valuable extensions that should be addressed. First, we only considered the quantity loss of FAPs. There are also quality loss issues and product freshness will change over time. In reality, consumers are paying more and more attention to the freshness of agricultural products. Therefore, it is also a meaningful research point to consider the changes in freshness. Second, in addition to the cost-sharing contract between the farmer and supplier, can other contracts (such as revenue-sharing and price discount contracts) also have the same or even better effect? We hope that this paper can inspire more research work on the contract farming supply chain with yield and demand uncertainties.

## Supporting information

S1 File (PDF)

## References

[1] Federgruen A., Lall U., Simsek A.S. (2019). Supply Chain Analysis of Contract Farming. Manufacturing & Service Operations Management, 21(2), 361–378. doi: 10.1287/msom.2018.0735

[2] Niu B., Jin D., Pu X. (2016). Coordination of channel members’ efforts and utilities in contract farming operations. European Journal of Operational Research, 255(3), 869–883. doi: 10.1016/j.ejor.2016.05.064

[3] Peng H., Pang T. (2019). Optimal strategies for a three-level contract-farming supply chain with subsidy. International Journal of Production Economics, 216, 274–286. doi: 10.1016/j.ijpe.2019.06.011

[4] Cai X., Chen J., Xiao Y., Xu X., Yu G. (2013). Fresh-product supply chain management with logistics outsourcing. Omega, 41(4), 752–765. doi: 10.1016/j.omega.2012.09.004

[5] Ye F., Lin Q., Li Y. (2020). Coordination for contract farming supply chain with stochastic yield and demand under CVaR criterion. Operational Research, 20(1), 369–397. doi: 10.1007/s12351-017-0328-3

[6] Yang L., Tang R., Chen K. (2017). Call, put and bidirectional option contracts in agricultural supply chains with sales effort. Applied Mathematical Modelling, 47, 1–16. doi: 10.1016/j.apm.2017.03.002

[7] Wang C., Chen X. (2015). Optimal ordering policy for a price-setting newsvendor with option contracts under demand uncertainty. International Journal of Production Research, 53(20), 6279–6293. doi: 10.1080/00207543.2015.1053577

[8] Liu G., Yang H., Dai R. (2020). Which contract is more effective in improving product greenness under different power structures: Revenue sharing or cost sharing?. Computers & Industrial Engineering, 148, 106701. doi: 10.1016/j.cie.2020.106701

[9] Song Z., He S. (2019). Contract coordination of new fresh produce three-layer supply chain. Industrial Management & Data Systems, 119(1), 148–169. doi: 10.1108/IMDS-12-2017-0559

[10] Chen J., Chen Y. (2021). The Impact of Contract Farming on Agricultural Product Supply in Developing Economies. Production and Operations Management. doi: 10.1111/poms.13382

[11] De A., Singh S.P. (2021). A resilient pricing and service quality level decision for fresh agri-product supply chain in post-COVID-19 era. International Journal of Logistics Management. doi: 10.1108/IJLM-02-2021-0117

[12] Moon I., Jeong Y.J., Saha S. (2020). Investment and coordination decisions in a supply chain of fresh agricultural products. Operational Research, 20. doi: 10.1007/s12351-018-0411-4

[13] Li M., He L., Yang G., Lian Z. (2022). Profit-Sharing Contracts for Fresh Agricultural Products Supply Chain Considering Spatio-Temporal Costs. Sustainability, 14(4), 2315. doi: 10.3390/su14042315

[14] Wan N., Li L., Wu X., Fan J. (2021). Coordination of a fresh agricultural product supply chain with option contract under cost and loss disruptions. PLOS ONE, 16(6), 0252960. doi: 10.1371/journal.pone.0252960 34106997 PMC8189505

[15] Zheng Q., Ieromonachou P., Fan T., Zhou L. (2017). Supply chain contracting coordination for fresh products with fresh-keeping effort. Industrial Management and Data Systems, 117(3), 538–559. doi: 10.1108/IMDS-04-2016-0139

[16] Wang C., Chen X. (2017). Option pricing and coordination in the fresh produce supply chain with portfolio contracts. Annals of Operations Research, 248(1), 471–491. doi: 10.1007/s10479-016-2167-7

[17] Niu B., Shen Z., Xie F. (2021). The value of blockchain and agricultural supply chain parties’ participation confronting random bacteria pollution. Journal of Cleaner Production, 319, 128579. doi: 10.1016/j.jclepro.2021.128579

[18] Liu Y., Ma D., Hu J., Zhang Z. (2021). Sales mode selection of fresh food supply chain based on blockchain technology under different channel competition. Computers & Industrial Engineering, 162, 107730. doi: 10.1016/j.cie.2021.107730

[19] Yan B., Wu X., Ye B., Zhang Y. (2017). Three-level supply chain coordination of fresh agricultural products in the internet of things. Industrial Management & Data Systems, 117(9), 1842–1865. doi: 10.1108/IMDS-06-2016-0245

[20] Wu X., Fan Z., Cao B. (2021). An analysis of strategies for adopting blockchain technology in the fresh product supply chain. International Journal of Production Research. doi: 10.1080/00207543.2021.1894497

[21] Ma X., Wang S., Sardar M.N., Islam, Liu X. (2019). Coordinating a three-echelon fresh agricultural products supply chain considering freshness-keeping effort with asymmetric information-science direct. Applied Mathematical Modelling, 67, 337–356. doi: 10.1016/j.apm.2018.10.028

[22] Cachon G. (2003). Supply chain coordination with contracts. Handbooks in Operations Research and Management Science, 11(11), 227–339. doi: 10.1016/S0927-0507(03)11006-7

[23] Zhao Y., Ma L., Xie G., Cheng T.C.E. (2013). Coordination of supply chains with bidirectional option contracts. European Journal of Operational Research, 229(2), 375–381. doi: 10.1016/j.ejor.2013.03.020

[24] Chen X., Luo J., Wang X., Yang D. (2020). Supply chain risk management considering put options and service level constraints. Computers & Industrial Engineering, 140, 106228. doi: 10.1016/j.cie.2019.106228

[25] Wang C., Chen J., Chen X. (2019). The impact of customer returns and bidirectional option contract on refund price and order decisions. European Journal of Operational Research, 274(1), 267–279. doi: 10.1016/j.ejor.2018.09.023

[26] Wan N., Chen X. (2019). The role of put option contracts in supply chain management under inflation. International Transactions in Operational Research, 26(4), 1451–1474. doi: 10.1111/itor.12372

[27] Shan R., Luo L., Kou R. (2021). Cost-sharing strategy for recycling and service investment in a closed-loop supply chain. RAIRO-Operations Research, 55(5), 2963–2990. doi: 10.1051/ro/2021143

[28] Chen X., Zhou J. (2021). The complexity analysis and chaos control in omni-channel supply chain with consumer migration and advertising cost sharing. Chaos Solitons & Fractals, 146(1), 110884. doi: 10.1016/j.chaos.2021.110884

[29] Yang S., Qian W. (2021). Effect of government subsidies on supply chain decision-making and coordination in the context of COVID-19. RAIRO-Operations Research, 55(3), 1885–1907. doi: 10.1051/ro/2021089

[30] Arani H.V., Rabbani M., Rafiei H. (2016). A revenue-sharing option contract toward coordination of supply chains. International Journal of Production Economics, 178, 42–56. doi: 10.1016/j.ijpe.2016.05.001

[31] Zhuo W., Shao L., Yang H. (2018). Mean-variance analysis of option contracts in a two-echelon supply chain. European Journal of Operational Research, 271(2), 535–547. doi: 10.1016/j.ejor.2018.05.033

[32] Esmaeili-Najafabadi E., Azad N., Nezhad M.S.F. (2021). Risk-averse supplier selection and order allocation in the centralized supply chains under disruption risks. Expert Systems with Applications, 175, 114691. doi: 10.1016/j.eswa.2021.114691

[33] Rockafellar R.T., Uryasev S. (2000). Optimization of conditional value-at-risk. Journal of Risk, 2(1), 1071–1074.

[34] Rockafellar R.T., Uryasev S. (2002). Conditional value-at-risk for general loss distributions. Journal of Banking & Finance, 26(7), 1443–1471. doi: 10.1016/S0378-4266(02)00271-6

[35] Fan Y., Feng Y., Shou Y. (2020). A risk-averse and buyer-led supply chain under option contract: CVaR minimization and channel coordination. International Journal of Production Economics, 219, 66–81. doi: 10.1016/j.ijpe.2019.05.021

[36] Zhao H., Wang H., Liu W., Song S., Liao Y. (2021). Supply chain coordination with a risk-averse retailer and the call option contract in the presence of a service requirement. Mathematics, 9(7), 787. doi: 10.3390/math9070787

[37] Shi Z., Cao E. (2020). Contract farming problems and games under yield uncertainty. Australian Journal of Agricultural and Resource Economics, 64(4), 1210–1238. doi: 10.1111/1467-8489.12400

