# Supplementary material for: Coordinating a three-level contract farming supply chain with option contracts considering risk-averse farmer and retailer
Source: PLoS One. 2023 Feb 24;18(2):e0279115. doi: 10.1371/journal.pone.0279115 (PMC9956081; doi:10.1371/journal.pone.0279115)
Supplement: S1 File — (PDF) [file pone.0279115.s001.pdf]

S1 Appendix

Appendix A: Basic notations

Table 1. Summary of basic notation.

Decision variables	
Q	Initial quantity ordered by the retailer
q	Option quantity ordered by the retailer
R	Production input of the farmer
Parameters	
w_f	Wholesale price of the supplier pays to the farmer
w_r	Wholesale price of the retailer pays to the supplier
p	Sale price of the retailer
e	Option exercise price
s_f	Initial product price in the farmer's spot market
φ	Farmer's replenishment cost sharing ratio
x	Random market demand, the mean is μ, the standard deviation is σ
y	Random output factor, the mean is μ_1, the standard deviation is σ_1
$o_1 \setminus o_2 \setminus o_3$	Call \ Put \ Bidirectional option price, where $o_3 > o_1$, $o_3 > o_2$
$c_s \setminus c_f$	Supplier's processing cost \ Farmer's planting cost
$\lambda_r \setminus \lambda_f$	Retailer's risk aversion coefficient \ Farmer's risk aversion coefficient
$\beta_r \setminus \beta_s$	Loss rate in distribution process from supplier to retailer \ from farmer to supplier
$\Pi \setminus E\Pi \setminus EU$	Profit function \ expected profit \ utility function
Superscript	
$CD \setminus DD$	Centralized \ decentralized supply chain
$CO \setminus PO \setminus BO$	Call \ put \ bidirectional option contract with wholesale price contract
$CC \setminus PC \setminus BC$	Call \ put \ bidirectional option contract with replenishment cost-sharing contract
$*$	Optimal expected profit, utility, or decision
Subscript	
$r \setminus s \setminus f$	Retailer \ Supplier \ Farmer

Appendix B: Proofs

Proof to Proposition 1. The first-order and second-order conditions with respect to Q_T and R_T, respectively, are
$\frac{\partial E\Pi_T^{CD}}{\partial Q_T} = p(1-\beta_r) - p(1-\beta_r)F[(1-\beta_r)Q_T] - c_s - \frac{s_f}{1-\beta_s}G(\frac{Q_T}{R_T(1-\beta_s)})$,
$\frac{\partial^2 E\Pi_T^{CD}}{\partial Q_T^2} = -p(1-\beta_r)^2 f[(1-\beta_r)Q_T] - \frac{s_f}{R_T(1-\beta_s)^2}g(\frac{Q_T}{R_T(1-\beta_s)})$,
$\frac{\partial E\Pi_T^{CD}}{\partial R_T} = -c_f + s_f\int_0^{\frac{Q_T}{R_T(1-\beta_s)}} yg(y)dy$, $\frac{\partial^2 E\Pi_T^{CD}}{\partial R_T^2} = -\frac{s_f Q_T^2}{R_T^3(1-\beta_s)^2}g(\frac{Q_T}{R_T(1-\beta_s)})$,
$\frac{\partial^2 E\Pi_T^{CD}}{\partial Q_T \partial R_T} = \frac{\partial^2 E\Pi_T^{CD}}{\partial R_T \partial Q_T} = \frac{s_f Q_T}{R_T^2(1-\beta_s)^2}g(\frac{Q_T}{R_T(1-\beta_s)})$. Then the Hessian matrix is
$H(Q_T, R_T) = \begin{bmatrix} \frac{\partial^2 E\Pi_T^{CD}}{\partial Q_T^2} & \frac{\partial^2 E\Pi_T^{CD}}{\partial Q_T \partial R_T} \\ \frac{\partial^2 E\Pi_T^{CD}}{\partial R_T \partial Q_T} & \frac{\partial^2 E\Pi_T^{CD}}{\partial R_T^2} \end{bmatrix}$. Obviously,
$|H(Q_T, R_T)| = \frac{s_f Q_T^2}{R_T^3(1-\beta_s)^2}g(\frac{Q_T}{R_T(1-\beta_s)})p(1-\beta_r)^2 f[(1-\beta_r)Q_T] > 0$, $\frac{\partial^2 E\Pi_T^{CD}}{\partial Q_T^2} < 0$, and $\frac{\partial^2 E\Pi_T^{CD}}{\partial R_T^2} < 0$. Thus, $H(Q_T, R_T)$ is a negative definite matrix, which implies that $E\Pi_T^{CD}$ jointly concave in (Q_T, R_T). The unique optimal Q_T^* and R_T^* should satisfy the first-order conditions. Hence, we have the proposition. □

Proof to Proposition 2. We denote $\alpha_{r1}^{DD} = -w_r(1-\beta_r)Q_0$, $\alpha_{r2}^{DD} = (p-w_r)(1-\beta_r)Q_0$, we know that $\alpha_{r1}^{DD} < \alpha_{r2}^{DD}$. When $\alpha_r^{DD} < \alpha_{r1}^{DD}$, $EU_r^{DD} = \alpha_r^{DD}$, there is $\frac{\partial EU_r^{DD}}{\partial \alpha_r^{DD}} = 1 > 0$. When $\alpha_{r1}^{DD} \le \alpha_r^{DD} < \alpha_{r2}^{DD}$,
$EU_r^{DD} = \alpha_r^{DD} - \frac{1}{\lambda_r}\int_0^{\frac{\alpha_r^{DD}-\alpha_{r1}^{DD}}{p}} (\alpha_r^{DD} - \alpha_{r1}^{DD} - px)f(x)dx$, there is
$\frac{\partial EU_r^{DD}}{\partial \alpha_r^{DD}} = 1 - \frac{1}{\lambda_r}F(\frac{\alpha_r^{DD}-\alpha_{r1}^{DD}}{p})$. From $\frac{\partial EU_r^{DD}}{\partial \alpha_r^{DD}} = 0$, we obtain $\alpha_r^{DD} = pF^{-1}(\lambda_r) + \alpha_{r1}^{DD}$.
When $\alpha_r^{DD} \ge \alpha_{r2}^{DD}$, the utility is $EU_r^{DD} =$
$\alpha_r^{DD} - \frac{1}{\lambda_r}\left[\int_0^{(1-\beta_r)Q_0} (\alpha_r^{DD} - \alpha_{r1}^{DD} - px)f(x)dx + \int_{(1-\beta_r)Q_0}^{+\infty} (\alpha_r^{DD} - \alpha_{r2}^{DD})f(x)dx\right]$.
Then, we can know that $\frac{\partial EU_r^{DD}}{\partial \alpha_r^{DD}} = 1 - \frac{1}{\lambda_r} < 0$. Thus, there are two cases:

(1) Suppose $pF^{-1}(\lambda_r) + \alpha_{r1}^{DD} < \alpha_{r2}^{DD}$. Thus $Q_0 > \frac{1}{1-\beta_r}F^{-1}(\lambda_r)$. Then, the optimal decision is $\alpha_r^{DD} = pF^{-1}(\lambda_r) + \alpha_{r1}^{DD}$. Substituting it into the utility function, there is $EU_r^{DD} = pF^{-1}(\lambda_r) - w_r(1-\beta_r)Q_0 - \frac{1}{\lambda_r}\int_0^{F^{-1}(\lambda_r)} p[F^{-1}(\lambda_r) - x]f(x)dx$. It's first derivative on Q_0 is $\frac{\partial EU_r^{DD}}{\partial Q_0} = -w_r(1-\beta_r) < 0$. Thus, we obtain $Q_0^* = \frac{1}{1-\beta_r}F^{-1}(\lambda_r)$, which contradicts with the result $Q_0 > \frac{1}{1-\beta_r}F^{-1}(\lambda_r)$. Thus $pF^{-1}(\lambda_r) + \alpha_{r1}^{DD} < \alpha_{r2}^{DD}$ does not hold.

(2) Suppose $pF^{-1}(\lambda_r) + \alpha_{r1}^{DD} \ge \alpha_{r2}^{DD}$. Thus $Q_0 \le \frac{1}{1-\beta_r}F^{-1}(\lambda_r)$. Then, the optimal decision is $\alpha_r^{DD} = \alpha_{r2}^{DD} = (p-w_r)(1-\beta_r)Q_0$. Substituting it into the utility function, there is $EU_r^{DD} = (p-w_r)(1-\beta_r)Q_0 - \frac{1}{\lambda_r}\left[\int_0^{(1-\beta_r)Q_0} p((1-\beta_r)Q_0 - x)f(x)dx\right]$. It's first and second derivatives on Q_0 are $\frac{\partial EU_r^{DD}}{\partial Q_0} = (p-w_r)(1-\beta_r) - \frac{p(1-\beta_r)}{\lambda_r}F[(1-\beta_r)Q_0]$ and $\frac{\partial^2 EU_r^{DD}}{\partial Q_0^2} = -\frac{p(1-\beta_r)^2}{\lambda_r}f((1-\beta_r)Q_0) < 0$. We can obtain $Q_0^* = \frac{1}{1-\beta_r}F^{-1}\left(\frac{\lambda_r(p-w_r)}{p}\right)$.

Hence, the retailer's optimal decisions are $\alpha_r^{DD} = (p-w_r)(1-\beta_r)Q_0$ and $Q_0^* = \frac{1}{1-\beta_r}F^{-1}\left(\frac{\lambda_r(p-w_r)}{p}\right)$.

Similar to the above process, we can obtain the farmer's optimal decisions. □

Proof to Proposition 3. (1) From Eq(1) and Eq(2), if $\lambda_f = 1$, then $\eta_1 = \eta$. Then, from the farmer and the supplier's optimal expected profit, there is
$(1-\beta_r)w_r > \frac{w_f}{1-\beta_s} + c_s > \frac{s_f G(\eta)}{1-\beta_s} + c_s$, i.e., $(1-\beta_r)w_r > c_s + c_\eta$. Thus
$Q_0^* = \frac{1}{1-\beta_r}F^{-1}\left(\frac{\lambda_r(p-w_r)}{p}\right) < \frac{1}{1-\beta_r}F^{-1}(1 - \frac{c_s+c_\eta}{p(1-\beta_r)}) = Q_T^*$. And from
$\eta = \frac{Q_T^*}{R_T^*(1-\beta_s)} = \frac{Q_0^*}{R_0^*(1-\beta_s)} = \eta_1$, there is $R_0^* < R_T^*$. (2) If $0 < \lambda_f < 1$, then $\eta_1 < \eta$, i.e.,

$\frac{Q_0^*}{R_0^*} < \frac{Q_T^*}{R_T^*}$. □

Proof to Proposition 4. We denote $\alpha_{r1}^{CO} = -w_r(1-\beta_r)Q_1 - o_1q_1$, $\alpha_{r2}^{CO} = (e-w_r)(1-\beta_r)Q_1 - o_1q_1$, $\alpha_{r3}^{CO} = (p-w_r)(1-\beta_r)Q_1 + (p-e)(1-\beta_r)q_1 - o_1q_1$, we know that $\alpha_{r1}^{CO} < \alpha_{r2}^{CO} < \alpha_{r3}^{CO}$. Thus, there are four cases:

(1) When $\alpha_r^{CO} \leq \alpha_{r1}^{CO}$, from Eq(4), there is $EU_r^{CO} = \alpha_r^{CO}$, and $\frac{\partial EU_r^{CO}}{\partial \alpha_r^{CO}} = 1 > 0$. So, the optimal decision is $\alpha_r^{CO} = \alpha_{r1}^{CO}$, and maximum utility is $EU_r^{CO} = -w_r(1-\beta_r)Q_1 - o_1q_1 \leq 0$. Then, only when $Q_1^* = q_1^* = 0$, the utility function becomes the largest. But only when $x = 0$, this case will appear, which is unreasonable. Thus, in this case, the retailer can not achieve maximum utility.

(2) When $\alpha_{r1}^{CO} < \alpha_r^{CO} \leq \alpha_{r2}^{CO}$, we have that $EU_r^{CO} = \alpha_r^{CO} - \frac{1}{\lambda_r}\int_0^{\frac{\alpha_r^{CO}-\alpha_{r1}^{CO}}{p}} (\alpha_r^{CO} - \alpha_{r1}^{CO} - px)f(x)dx$, and $\frac{\partial EU_r^{CO}}{\partial \alpha_r^{CO}} = 1 - \frac{1}{\lambda_r}F(\frac{\alpha_r^{CO}-\alpha_{r1}^{CO}}{p})$. From $\frac{\partial EU_r^{CO}}{\partial \alpha_r^{CO}} = 0$, the optimal decision is $\alpha_r^{CO} = pF^{-1}(\lambda_r) + \alpha_{r1}^{CO}$. Substituting it into the utility function is $EU_r^{CO} = pF^{-1}(\lambda_r) - w_r(1-\beta_r)Q_1 - o_1q_1 - \frac{1}{\lambda_r}\int_0^{F^{-1}(\lambda_r)} p[F^{-1}(\lambda_r) - x]f(x)dx$. We can know that only when $0 < x \leq F^{-1}(\lambda_r) < (1-\beta_r)Q_1$, this scenario will appear. We have that $\frac{\partial EU_r^{CO}}{\partial Q_1} = -w_r(1-\beta_r) < 0$ and $\frac{\partial EU_r^{CO}}{\partial q_1} = -o_1 < 0$, we can know that the market demand can be met when $Q_1^* = F^{-1}(\lambda_r)$. And the retailer does not need to purchase call options and can not achieve the maximum utility.

(3) When $\alpha_{r2}^{CO} < \alpha_r^{CO} \leq \alpha_{r3}^{CO}$, the utility is $EU_r^{CO} = \alpha_r^{CO} - \frac{1}{\lambda_r}\int_0^{(1-\beta_r)Q_1} (\alpha_r^{CO} - \alpha_{r1}^{CO} - px)f(x)dx - \frac{1}{\lambda_r}\int_{(1-\beta_r)Q_1}^{\frac{\alpha_r^{CO}-\alpha_{r2}^{CO}}{p-e}} (\alpha_r^{CO} - \alpha_{r2}^{CO} - (p-e)x)f(x)dx$. We know that $\frac{\partial EU_r^{CO}}{\partial \alpha_r^{CO}} = 1 - \frac{1}{\lambda_r}F(\frac{\alpha_r^{CO}-\alpha_{r2}^{CO}}{p-e})$. From $\frac{\partial EU_r^{CO}}{\partial \alpha_r^{CO}} = 0$, we get the optimal decision $\alpha_r^{CO} = (p-e)F^{-1}(\lambda_r) + \alpha_{r2}^{CO}$. Substituting it into the utility function, then $EU_r^{CO} = (p-e)F^{-1}(\lambda_r) + (e-w_r)(1-\beta_r)Q_1 - o_1q_1 - \frac{1}{\lambda_r}p\int_0^{(1-\beta_r)Q_1} F(x)dx - \frac{1}{\lambda_r}(p-e)\int_{(1-\beta_r)Q_1}^{F^{-1}(\lambda_r)} F(x)dx$. We can know that only when $0 < x \leq F^{-1}(\lambda_r) < (1-\beta_r)(Q_1+q_1)$, this scenario will appear. We have that $\frac{\partial EU_r^{CO}}{\partial Q_1} = (e-w_r)(1-\beta_r) - \frac{1}{\lambda_r}e(1-\beta_r)F((1-\beta_r)Q_1)$ and $\frac{\partial EU_r^{CO}}{\partial q_1} = -o_1 < 0$. From $\frac{\partial EU_r^{CO}}{\partial Q_1} = 0$ and the range of x, we can obtain that $Q_1^* = \frac{1}{1-\beta_r}F^{-1}(\frac{\lambda_r(e-w_r)}{e})$. Since $Q_1 + q_1$ equal to the maximum market demand $F^{-1}(\lambda_r)$, then $q_1^* = F^{-1}(\lambda_r) - \frac{1}{1-\beta_r}F^{-1}(\frac{\lambda_r(e-w_r)}{e})$. At this time, the retailer purchased a part of the call options and only met a part of demand, so he can not achieve the maximum utility.

(4) When $\alpha_r^{CO} \geq \alpha_{r3}^{CO}$, the utility function is $EU_r^{CO} = \alpha_r^{CO} - \frac{1}{\lambda_r}\int_0^{(1-\beta_r)Q_1} (\alpha_r^{CO} - \alpha_{r1}^{CO} - px)f(x)dx - \frac{1}{\lambda_r}\int_{(1-\beta_r)Q_1}^{(1-\beta_r)(Q_1+q_1)} (\alpha_r^{CO} - \alpha_{r2}^{CO} - (p-e)x)f(x)dx - \frac{1}{\lambda_r}\int_{(1-\beta_r)(Q_1+q_1)}^{+\infty} (\alpha_r^{CO} - \alpha_{r3}^{CO})f(x)dx$. We know that $\frac{\partial EU_r^{CO}}{\partial \alpha_r^{CO}} = 1 - \frac{1}{\lambda_r} < 0$, so we can obtain that the optimal threshold of loss is $\alpha_r^{CO} = \alpha_{r3}^{CO}$. Substituting it into the utility function is $EU_r^{CO} = (p-e)(1-\beta_r)q_1 + (p-w_r)(1-\beta_r)Q_1 - o_1q_1 - \frac{1}{\lambda_r}\left[p\int_0^{(1-\beta_r)Q_1} F(x)dx + (p-e)\int_{(1-\beta_r)Q_1}^{(1-\beta_r)(Q_1+q_1)} F(x)dx\right]$. The first-order and second-order conditions of EU_r^{CO} with respect to Q_1 and q_1, respectively, are

$\frac{\partial EU_r^{CO}}{\partial Q_1} = (p-w_r)(1-\beta_r) - \frac{1-\beta_r}{\lambda_r}[eF((1-\beta_r)Q_1) + (p-e)F((1-\beta_r)(Q_1+q_1))]$,

$\frac{\partial^2 EU_r^{CO}}{\partial Q_1^2} = -\frac{(1-\beta_r)^2}{\lambda_r}[ef((1-\beta_r)Q_1) + (p-e)f((1-\beta_r)(Q_1+q_1))]$,

$\frac{\partial EU_r^{CO}}{\partial q_1} = (p-e)(1-\beta_r) - o_1 - \frac{(p-e)(1-\beta_r)}{\lambda_r}F((1-\beta_r)(Q_1+q_1))$,

$\frac{\partial^2 EU_r^{CO}}{\partial q_1^2} = -\frac{(p-e)(1-\beta_r)^2}{\lambda_r}f((1-\beta_r)(Q_1+q_1))$,

$\frac{\partial^2 EU_r^{CO}}{\partial Q_1 \partial q_1} = \frac{\partial^2 EU_r^{CO}}{\partial q_1 \partial Q_1} = -\frac{(p-e)(1-\beta_r)^2}{\lambda_r} f((1-\beta_r)(Q_1+q_1))$. Then the Hessian matrix is $H(Q_1, q_1) = \begin{bmatrix} \frac{\partial^2 EU_r^{CO}}{\partial Q_1^2} & \frac{\partial^2 EU_r^{CO}}{\partial Q_1 \partial q_1} \\ \frac{\partial^2 EU_r^{CO}}{\partial q_1 \partial Q_1} & \frac{\partial^2 EU_r^{CO}}{\partial q_1^2} \end{bmatrix}$. It is easy to check that $|H(Q_1, q_1)| = \frac{e(p-e)(1-\beta_r)^4}{\lambda_r^2} f((1-\beta_r)Q_1) f((1-\beta_r)(Q_1+q_1)) > 0$, $\frac{\partial^2 EU_r^{CO}}{\partial Q_1^2} < 0$, and $\frac{\partial^2 EU_r^{CO}}{\partial q_1^2} < 0$. Thus, $H(Q_1, q_1)$ is a negative definite matrix, which implies that EU_r^{CO} jointly concave in (Q_1, q_1). The unique optimal Q_1^* and q_1^* should satisfy the first-order conditions. In addition, from $q_1^* > 0$, there is $\frac{\lambda_r((p-e)(1-\beta_r)-o_1)}{(p-e)(1-\beta_r)} > \frac{\lambda_r((e-w_r)(1-\beta_r)+o_1)}{e(1-\beta_r)}$, i.e., $(p-e)(1-\beta_r)w_r > po_1$.

Therefore, the retailer's optimal decision is $\alpha_r^{CO} = \alpha_{r3}^{CO} = (p-w_r)(1-\beta_r)Q_1 + (p-e)(1-\beta_r)q_1 - o_1 q_1$, Q_1^*, and q_1^*.

Similar to the Proof of Proposition 2, we can obtain the farmer's optimal decision. □

Proof to Proposition 5. (1) From Eq(1) and Eq(5), we know that when $\lambda_f = 1$, $\eta_1 = \eta$, then from $Q_1^* + q_1^* = Q_T^*$, we have that $o_1 = \frac{(p-e)[c_s + c_\eta - p(1-\beta_r)(1-\lambda_r)]}{\lambda_r p}$, and from $w_r(p-e)(1-\beta_r) > po_1$ and $e(1-\beta_r) + o_1 > w_r(1-\beta_r)$, we have that $\frac{w_r(1-\beta_r)-o_1}{1-\beta_r} < e < \frac{p[w_r(1-\beta_r)-o_1]}{w_r(1-\beta_r)}$. From $\eta = \frac{Q_T^*}{R_T^*(1-\beta_s)} = \frac{Q_1^*+q_1^*}{R_1^*(1-\beta_s)} = \eta_1$, we know that $R_1^* = R_T^*$. (2) We know that when $0 < \lambda_f < 1$, $\eta_1 < \eta$, thus for any o_1 and e, that is $\frac{Q_1^*+q_1^*}{R_1^*} < \frac{Q_T^*}{R_T^*}$. □

Proof to Proposition 6. In the call option contract with replenishment cost-sharing contract, from Eq(1) and Eq(7), we know that when $\varphi = \lambda_f$, $\eta_1^C = \eta$. Then from $Q_1^* + q_1^* = Q_T^*$, we have that $o_1 = \frac{(p-e)[c_s + c_\eta - p(1-\beta_r)(1-\lambda_r)]}{\lambda_r p}$, and from $w_r(p-e)(1-\beta_r) > po_1$ and $e(1-\beta_r) + o_1 > w_r(1-\beta_r)$, we have that $\frac{w_r(1-\beta_r)-o_1}{1-\beta_r} < e < \frac{p[w_r(1-\beta_r)-o_1]}{w_r(1-\beta_r)}$. From $\eta = \frac{Q_T^*}{R_T^*(1-\beta_s)} = \frac{Q_1^*+q_1^*}{R_1^{C*}(1-\beta_s)} = \eta_1^C$, we know that $R_1^{C*} = R_T^*$. □

Proof to Corollary 1. (1) From Proposition 4, we have that $\frac{\partial Q_1^*}{\partial \lambda_r} = \frac{(e-w_r)(1-\beta_r)+o_1}{e(1-\beta_r)^2 f((1-\beta_r)Q_1^*)} > 0$, and $Q_1^* + q_1^* = \frac{1}{1-\beta_r} F^{-1}(\frac{\lambda_r((p-e)(1-\beta_r)-o_1)}{(p-e)(1-\beta_r)})$, then we have that $\frac{\partial (Q_1^*+q_1^*)}{\partial \lambda_r} = \frac{(p-e)(1-\beta_r)-o_1}{(p-e)(1-\beta_r)^2 f((1-\beta_r)(Q_1^*+q_1^*))} > 0$. From $\eta_1 = \frac{Q_1^*+q_1^*}{R_1^*(1-\beta_s)}$, we have that $\frac{\partial R_1^*}{\partial \lambda_r} = \frac{1}{\eta_1(1-\beta_s)} * \frac{\partial (Q_1^*+q_1^*)}{\partial \lambda_r} > 0$. From Eq(5), we have that $\frac{\partial R_1^*}{\partial \lambda_f} = -\frac{c_f R_1^{*2}(1-\beta_s)}{s_f \eta_1 g(\eta_1)(Q_1^*+q_1^*)} < 0$. (2) Similar to above, we have that

$\frac{\partial Q_1^*}{\partial o_1} = \frac{\lambda_r}{e(1-\beta_r)^2 f((1-\beta_r)Q_1^*)} > 0$, $\frac{\partial (Q_1^*+q_1^*)}{\partial o_1} = -\frac{\lambda_r}{(p-e)(1-\beta_r)^2 f((1-\beta_r)(Q_1^*+q_1^*))} < 0$,

$\frac{\partial q_1^*}{\partial o_1} = \frac{\partial (Q_1^*+q_1^*)}{\partial o_1} - \frac{\partial Q_1^*}{\partial o_1} < 0$, $\frac{\partial R_1^*}{\partial o_1} = \frac{1}{\eta_1(1-\beta_s)} * \frac{\partial (Q_1^*+q_1^*)}{\partial o_1} < 0$,

$\frac{\partial Q_1^*}{\partial e} = \frac{\lambda_r[w_r(1-\beta_r)-o_1]}{e^2(1-\beta_r)^2 f((1-\beta_r)Q_1^*)} > 0$, $\frac{\partial (Q_1^*+q_1^*)}{\partial e} = -\frac{\lambda_r o_1}{(p-e)^2(1-\beta_r)^2 f((1-\beta_r)(Q_1^*+q_1^*))} < 0$,

$\frac{\partial q_1^*}{\partial e} = \frac{\partial (Q_1^*+q_1^*)}{\partial e} - \frac{\partial Q_1^*}{\partial e} < 0$, $\frac{\partial R_1^*}{\partial e} = \frac{1}{\eta_1(1-\beta_s)} * \frac{\partial (Q_1^*+q_1^*)}{\partial e} < 0$. □

Proof to Proposition 7. The proof process is similar to Proposition 4. So, we omit it here. □

Proof to Proposition 8. The proof process is similar to Proposition 5. So, we omit it here. □

Proof to Proposition 9. The proof process is similar to Proposition 6. So, we omit it here. □

Proof to Corollary 2. The proof process is similar to Corollary 1. So, we omit it here. □

Proof to Proposition 10. The proof process is similar to Proposition 4. So, we omit it here. □

Proof to Proposition 11. The proof process is similar to Proposition 5. So, we omit it here. □

Proof to Proposition 12. The proof process is similar to Proposition 6. So, we omit it here. □

Proof to Corollary 3. The proof process is similar to Corollary 1. So, we omit it here. □

Proof to Proposition 13. From $p(1-\beta_r) > e(1-\beta_r)+o_3 > e(1-\beta_r)+o_1 > w_r(1-\beta_r) > e(1-\beta_r)-o_2 > e(1-\beta_r)-o_3$, we have that $o_3 > o_1$, $o_3 > o_2$ and $w_r - \frac{o_1}{1-\beta_r} < e < \min\{p - \frac{o_3}{1-\beta_r}, w_r + \frac{o_2}{1-\beta_r}\}$. From Proposition 4, 7, and 10, we have that when $o_3 > 2o_2 - (e-w_r)(1-\beta_r)$, $Q_3^* + q_3^* < Q_2^*$; When $o_3 > 2o_1 + (e-w_r)(1-\beta_r)$, $Q_3^* + q_3^* < Q_1^* + q_1^*$; When $o_2 > o_1 + (e-w_r)(1-\beta_r)$, $Q_1^* + q_1^* > Q_2^*$. Then from $\eta_1 = \frac{Q_1^*+q_1^*}{R_1^*(1-\beta_s)} = \frac{Q_2^*}{R_2^*(1-\beta_s)} = \frac{Q_3^*+q_3^*}{R_3^*(1-\beta_s)}$, $\eta_1^C = \frac{Q_1^*+q_1^*}{R_1^{C*}(1-\beta_s)} = \frac{Q_2^*}{R_2^{C*}(1-\beta_s)} = \frac{Q_3^*+q_3^*}{R_3^{C*}(1-\beta_s)}$, and Eq(3), (6), (10), (8), (12), (16), we know that the size comparison of the farmer's optimal production input and optimal expected profit are consistent with the retailer's optimal total order quantity. Hence, combining the above conditions, we have the proposition. □

Proof to Proposition 14.

From $Q_1^* + q_1^* = Q_2^*$, that is $\frac{1}{1-\beta_r}F^{-1}[\frac{\lambda_r((p-e)(1-\beta_r)-o_1)}{(p-e)(1-\beta_r)}] = \frac{1}{1-\beta_r}F^{-1}[\frac{\lambda_r((p-w_r)(1-\beta_r)-o_2)}{(p-e)(1-\beta_r)}]$, we have that $o_2 = (e-w_r)(1-\beta_r) + o_1$. From $Q_1^* + q_1^* = Q_3^* + q_3^*$, that is $\frac{1}{1-\beta_r}F^{-1}[\frac{\lambda_r((p-e)(1-\beta_r)-o_1)}{(p-e)(1-\beta_r)}] = \frac{1}{1-\beta_r}F^{-1}[\frac{\lambda_r((2p-e-w_r)(1-\beta_r)-o_3)}{2(p-e)(1-\beta_r)}]$, we have that $o_3 = (e-w_r)(1-\beta_r) + 2o_1$. So when $o_2 = (e-w_r)(1-\beta_r) + o_1$ and $o_3 = (e-w_r)(1-\beta_r) + 2o_1$, $Q_1^* + q_1^* = Q_2^* = Q_3^* + q_3^*$.

Then, from $q_2^* = Q_2^* - \frac{1}{1-\beta_r}F^{-1}[\frac{\lambda_r o_2}{e(1-\beta_r)}] = Q_1^* + q_1^* - \frac{1}{1-\beta_r}F^{-1}[\frac{\lambda_r o_2}{e(1-\beta_r)}]$ and $\frac{1}{1-\beta_r}F^{-1}[\frac{\lambda_r o_2}{e(1-\beta_r)}] = \frac{1}{1-\beta_r}F^{-1}[\frac{\lambda_r((e-w_r)(1-\beta_r)+o_1)}{e(1-\beta_r)}] = Q_1^*$, we have that $q_1^* = q_2^*$, from $2q_3^* = Q_3^* + q_3^* - (Q_3^* - q_3^*) = Q_2^* - (Q_3^* - q_3^*)$ and $Q_3^* - q_3^* = \frac{1}{1-\beta_r}F^{-1}[\frac{\lambda_r((e-w_r)(1-\beta_r)+o_3)}{2e(1-\beta_r)}] = \frac{1}{1-\beta_r}F^{-1}[\frac{\lambda_r((e-w_r)(1-\beta_r)+o_1)}{e(1-\beta_r)}] = \frac{1}{1-\beta_r}F^{-1}[\frac{\lambda_r o_2}{e(1-\beta_r)}]$, we have that $2q_3^* = q_2^*$, thus $q_1^* = q_2^* = 2q_3^*$.

From $Q_1^* + q_1^* = Q_2^* = Q_3^* + q_3^*$ and $\eta_1 = \frac{Q_1^*+q_1^*}{R_1^*(1-\beta_s)} = \frac{Q_2^*}{R_2^*(1-\beta_s)} = \frac{Q_3^*+q_3^*}{R_3^*(1-\beta_s)}$ or $\eta_1^C = \frac{Q_1^*+q_1^*}{R_1^{C*}(1-\beta_s)} = \frac{Q_2^*}{R_2^{C*}(1-\beta_s)} = \frac{Q_3^*+q_3^*}{R_3^{C*}(1-\beta_s)}$, we have that $R_1^* = R_2^* = R_3^*$ and $R_1^{C*} = R_2^{C*} = R_3^{C*}$.

Combining the above conditions and conclusions, we can obtain Proposition 14(2)(3)(4). Hence, we have the proposition. □

Proof to Proposition 15. When the supply chain can be fully coordinated, in the call option contract, we know that is Proposition 5(1) and Proposition 6. According to them, we can obtain that $E\Pi_f^{CO*} = [w_f - s_f G(\eta)]\frac{Q_T^*}{1-\beta_s}$ and $E\Pi_f^{CC*} = [w_f - \lambda_f s_f G(\eta) - \frac{(1-\lambda_f)c_f}{\eta}]\frac{Q_T^*}{1-\beta_s}$. Thus, if $s_f > \frac{c_f}{\eta G(\eta)}$, then $E\Pi_f^{CO*} < E\Pi_f^{CC*}$; If $c_f < s_f < \frac{c_f}{\eta G(\eta)}$, then $E\Pi_f^{CO*} > E\Pi_f^{CC*}$. According to $E\Pi_T^{CC*} = E\Pi_T^{CO*} = E\Pi_T^{CD*}$ and $E\Pi_r^{CC*} = E\Pi_r^{CO*}$, we can know that if $s_f > \frac{c_f}{\eta G(\eta)}$, then $E\Pi_s^{CO*} > E\Pi_s^{CC*}$; If $c_f < s_f < \frac{c_f}{\eta G(\eta)}$, then $E\Pi_s^{CO*} < E\Pi_s^{CC*}$. Similarly, we can proof the scenarios of put option contract and bidirectional option contract. □
